# The long noncoding RNA *FRILAIR* regulates strawberry fruit ripening by functioning as a noncanonical target mimic

**DOI:** 10.1371/journal.pgen.1009461

**Published:** 2021-03-19

**Authors:** Yajun Tang, Zhipeng Qu, Jiajun Lei, Reqing He, David L. Adelson, Youlin Zhu, Zhenbiao Yang, Dong Wang

**Affiliations:** 1 College of Horticulture, Fujian Agriculture and Forestry University, Fuzhou, China; 2 Key Laboratory of Molecular Biology and Gene Engineering in Jiangxi Province, College of Life Science, Nanchang University, Nanchang, China; 3 FAFU-UCR Joint Center for Horticultural Biology and Metabolomics, Haixia Institute of Science and Technology, Fujian Agriculture and Forestry University, Fuzhou, China; 4 Department of Molecular and Biomedical Science, School of Biological Sciences, The University of Adelaide, Adelaide, Australia; 5 College of Horticulture, Shenyang Agricultural University, Shenyang, China; Gregor Mendel Institute of Molecular Plant Biology, AUSTRIA

## Abstract

Long noncoding RNAs (lncRNAs) are emerging as important regulators in plant development, but few of them have been functionally characterized in fruit ripening. Here, we have identified 25,613 lncRNAs from strawberry ripening fruits based on RNA-seq data from poly(A)-depleted libraries and rRNA-depleted libraries, most of which exhibited distinct temporal expression patterns. A novel lncRNA, *FRILAIR* harbours the miR397 binding site that is highly conserved in diverse strawberry species. *FRILAIR* overexpression promoted fruit maturation in the Falandi strawberry, which was consistent with the finding from knocking down miR397, which can guide the mRNA cleavage of both *FRILAIR* and *LAC11a* (encoding a putative laccase-11-like protein). Moreover, *LAC11a* mRNA levels were increased in both *FRILAIR* overexpressing and miR397 knockdown fruits, and accelerated fruit maturation was also found in *LAC11a* overexpressing fruits. Overall, our study demonstrates that *FRILAIR* can act as a noncanonical target mimic of miR397 to modulate the expression of *LAC11a* in the strawberry fruit ripening process.

## Author summary

Strawberry is a non-climacteric fruit with a typical ripening process, which is cultivated worldwide for its fruit. The alteration of fruit colour change is commonly used as an intuitive and commercial indicator to determine strawberry ripeness. Long noncoding RNAs (lncRNAs) are emerging as important regulators in plant development, and have been widely identified in strawberry. However, few of them have been functionally characterized in strawberry fruit ripening. Here, we performed transcriptome sequencing on fruits of the diploid strawberry *F*. *vesca* (Hawaii 4) at three developmental stages. An intergenic lncRNA, *FRILAIR* (FRUIT RIPENING-RELATED LONG INTERGENIC RNA) harbours a miR397 binding site that is highly conserved in diverse strawberry species. Accumulation of *FRILAIR* can release repression of *LAC11a* that is the miR397 target, which subsequently promotes expressions of genes involved in anthocyanin biosynthesis pathway, leading to the acceleration of strawberry fruit ripening. Our study demonstrates that *FRILAIR* modulates strawberry fruit ripening through fine-tuning miRNA activity, suggesting that lncRNAs provide a valuable resource for the manipulation of the gene regulatory network responsible for strawberry fruit ripening.

## Introduction

Long non-coding RNA (lncRNA) is a wide-spread subset of ncRNAs in animal and plants. In mammals, lncRNAs can function as important regulators in alternative splicing of endogenous pre-mRNAs [1], chromatin state control [2], transcriptional [3] and post-transcriptional gene regulation [4]. In addition, transcriptome-wide analyses have revealed the important regulatory roles of lncRNAs in a set of plant biological processes, such as ribosome-associated noncoding RNAs regulating mRNA abundance and translation in *Arabidopsis thaliana* [5], lncRNA16397 involved in the response of tomato to *P*. *infestans* infection [6] and lncRNAs associated with *Fragaria vesca* (*F*. *vesca*) reproductive development [7]. Although many lncRNAs have been identified in diverse plant species, their biological functions are still largely unknown.

Fruit ripening is a complex biological process that changes metabolic and physiological traits. In pear, miR397a expression was significantly changed between various fruit developmental stages [8]. Overexpressing OsmiR397 could enlarge grain size and promote panicle branching by downregulating its target *OsLAC* [9]. Emerging evidence suggests that lncRNAs play important roles in fleshy fruit ripening. In tomato (*Solanum lycopersicum L*. cv. Micro-Tom) fruit, knocking down lncRNA1459 and lncRNA1840 by a virus-induced gene silencing system (VIGS) delayed ripening [10], and silencing of two lncRNAs (LNC1 and LNC2) *in vivo* affected anthocyanin biosynthesis during fruit ripening in sea buckthorn [11]. Strawberry is a non-climacteric fruit with a typical ripening process, and alteration of strawberry fruit colour change is commonly used as an intuitive and commercial indicator to determine strawberry ripeness [12–14]. Previous studies have shown that protein-coding genes associated with abscisic acid (ABA) biosynthesis and catabolism are involved in strawberry fruit ripening, such as *FaNCED1* [15] and *FveCYP707A4a* [16]. LncRNAs have also been identified in the diploid woodland strawberry during flower and fruit development through transcriptome analyses [7], but their potential roles in fruit ripening have not been systematically studied.

To explore the contributions of lncRNAs to strawberry fruit ripening, we performed RNA-seq on fruits of the diploid strawberry *F*. *vesca* at three developmental stages. Here, we report the identification of 25,613 lncRNAs from ribosomal RNA (rRNA)-depleted RNA sequencing along with poly(A)-depleted RNA sequencing, and show that some exhibited temporal expression specificity. We characterised a novel lncRNA, *FRILAIR* (*FRUIT RIPENING-RELATED LONG INTERGENIC RNA*), containing a miR397 binding site, that when over-expressed promoted fruit maturation in the octoploid strawberry Falandi (*Fragaria* × *ananassa* Duch.), which was consistent with the finding that knocking down miR397, that guides the cleavage of *FRILAIR* and *LAC11a* transcripts also promoted fruit ripening. Expression levels of *LAC11a*, that encodes a putative laccase-11-like protein, were increased in both *FRILAIR* over-expressing and miR397 knock down strawberry fruits, and accelerated fruit maturation was observed in *LAC11a* over-expressing fruits. By co-expressing *FRILAIR* and miR397 at different concentration combination in strawberry fruits, we found that the endogenous *LAC11a* expression was strikingly decreased in fruits with the higher ratio of miR397 to *FRILAIR*, and an opposite expression trend of *LAC11a* was present in fruits with the lower ratio of miR397 to *FRILAIR*. Further analyses revealed that *LAC11a* promotes expressions of genes involved in anthocyanin biosynthesis pathway, leading to the acceleration of fruit maturation in strawberry. Taken together, our results indicate that *FRILAIR* can act as a noncanonical target mimic of miR397 to regulate the expression of its target gene *LAC11a* during strawberry fruit ripening.

## Material and methods

### Plant materials and growth conditions

Strawberry plants *F*. *vesca* (Hawaii 4) were grown at 20°C-23°C in a greenhouse (60–70% relative humidity, 10-h light, 14-h dark cycle), and Falandi (*Fragaria* × *ananassa* Duch.) were grown at a greenhouse in Qujing (Yunnan Province, China). Fruits were collected, then immediately snap frozen in liquid nitrogen and stored at -80°C.

### Nuclear and cytoplasmic RNA extraction

Nuclear and Cytoplasmic RNA extraction of strawberry fruits was performed according to the previous study [17]. *U6* and *tRNA* were used as internal controls for nuclear and cytoplasmic extracts respectively. Primers used in this study are listed in S9 Table.

### Plasmid construction

To generate the *FRILAIR* overexpression vector, we amplified and cloned the full-length cDNA of *FRILAIR* (1,520 bp) into the pCAMBIA1300 backbone vector using *Kpn*I and *Xba*I under the control of the 2× 35S promoter. The same strategy was applied to construct the *LAC11a* (1,689 bp) and miR397 (622 bp) overexpression vector. To generate *FRILAIR mut1*, Fmimic1 and Fmimic2, oligonucleotide-directed mutagenesis in the miR397 target site was constructed by two-step PCR assays using chimeric primer pairs containing the overlapped linker. *FRILAIR mut2* and *FRILAIR mut3* vectors were generated by using the same strategy, and a 21 bp deletion spanning the miRNA397 target site sequence and six mismatches in the miR397 target site were produced by overlapping two PCR amplicons, respectively. For *GFP*:*FRILAIR* construct, a GFP fragment was cloned at the upstream of *FRILAIR* in pCAMBIA1300. Meanwhile, *GFP*:*FRILAIR mut2* and *GFP*:*FRILAIR mut3* vectors were generated through using the same strategy. For miR397 knockdown (miR397i), *FRILAIR* knockdown (*FRILAIR* KD) and *LAC11a* knockdown (*LAC11a* KD) vectors, *Cas13b* was synthesized according to an earlier study [18], then a fragment coding for the nuclear localization signal (NLS) together with *Cas13b* were cloned into the pCAMBIA1300 backbone vector using *Xho*I and *EcoR*I under the control of the 2× 35S promoter. In addition, a DNA fragment comprised of a FveU6-2 promoter [19], two *Bsa*I sites and the sgRNA scaffold of Cas13b was produced by overlapping PCR. The resultant DNA fragment was inserted upstream of the Cas13b cassette by *Hind*III and *Pst*I, leading to the vector pFveCas13b. We synthesized the 30 bp sgRNA target sequence, and annealed it on a PCR machine. The annealed oligo adaptors were inserted into the *Bsa*I digested pFveCas13b vector, and Sanger sequencing was performed to confirm vector sequences. Primers used for vector and sgRNA constructions are listed in S9 Table.

### RNA extraction, RNA sequencing and qRT-PCR

Total RNA was extracted from strawberry fruit tissues using TRIzol (Invitrogen, Carlsbad, USA) according to the manufacturer’s instructions. RNA-seq libraries made from rRNA-depleted RNA and poly(A)-depleted RNA were prepared as previously described [20], and then sequenced on the Illumina HiSeq 2500 platform. Five to ten strawberry fruit tissues from several independent plants were combined to form one biological replicate, and all experiments were carried out with three biological replicates. Strawberry cDNA was synthesized from total RNA using the TransScript All-in-One First-Strand cDNA Synthesis SuperMix (Transgen Biotech, Beijing, China), and real-time quantitative PCR (qRT-PCR) was performed using TB Green Premix Ex Taq II (Takara Bio, Tokyo, Japan). *U6*, *ACTIN* and *GAPDH* were used as internal controls, respectively. Primer sequences used for qRT-PCR in this study are shown in S9 Table.

### LncRNA-miRNA-gene interaction network analysis

Sequences of all mature *F*. *vesca* miRNAs were extracted from miRBase [21]. Potential targets of *F*. *vesca* miRNAs in genes and lncRNAs were predicted using TAPIR (http://bioinformatics.psb.ugent.be/webtools/tapir/) and psRNATarget (https://plantgrn.noble.org/psRNATarget/) with default settings. Only consistent predictions from both tools were considered as miRNA-target pairs and used for analysis.

### Co-expression networks analysis

Adaptor and low quality sequences from raw reads were trimmed using Trimmomatic (v0.36) with following settings: LEADING:3 TRAILING:3 SLIDINGWINDOW:4:15 MINLEN:20. Clean reads were mapped against *F*. *vesca*_V4.0 genome using STAR (2.7.1a) with following parameters:—outFilterMismatchNmax 10—outFilterMismatchNoverLmax 0.05—seedSearchStartLmax 30. Raw read count table for all identified lncRNAs (25,124) and reference genes (28,588) were generated using featureCounts in Rsubread package. Then only genes/lncRNAs with more than 10 reads in more than 60% of the total number of samples (more than 43 out of 72 samples in this study) were kept for co-expression analysis. Batch effect from filtered read count table was removed using RUVseq package [22], and then read counts were normalised using VST (Variance stabilizing Transformation) method. WGCNA was used to do co-expression analysis for filtered lncRNAs/genes [23]. Public datasets including PRJEB12420, SRP111905 and GSE113084 were used for co-expression network analysis.

### *Agrobacterium tumefaciens*-mediated transient transformation in strawberry fruits

Vectors were transformed into the *Agrobacterium tumefaciens* strain GV3101 for transient transformation in strawberry fruits by using the infection method described previously [15,24]. In brief, the agrobacterium harbouring the corresponding vector was grown in liquid LB medium until OD600 reached 0.8–1.0, then the culture was spun down and re-suspended in the infiltration buffer (10 mM MES, 150 μM AS, 10 mM MgC1_2_) to reach an OD600 of 0.8. Fruits at big green stage were injected with a 10 ml syringe. To examine the effects of *FRILAIR* overexpression (*FRILAIR* OE), *LAC11a* overexpression (*LAC11a* OE) and miR397 overexpression (miR397 OE), *FRILAIR* KD, *LAC11a* KD and miR397i on strawberry fruit ripening, we selected Falandi fruits in big green stage for transient transformation. Transcriptional levels of *FRILAIR*, miR397 and *LAC11a* were evaluated at five days after infection. For each vector, 5–10 fruits were collected from at least five strawberry plants under *Agrobacterium tumefaciens*-mediated transient transformation for analysis. Each experiment was repeated three times.

### RLM-RACE

RNA ligase-mediated rapid amplification of cDNA ends (RLM-RACE) was performed according to an earlier study [25], and *FRILAIR* OE and *LAC11a* OE fruits were used in this analysis. Primers used in nested PCR for *FRILAIR* and *LAC11a* are listed in S9 Table. Resultant PCR amplicons were gel purified and cloned into the pEASY Cloning Kit (Transgen Biotech, Beijing, China) for sequencing.

### Measurement on total anthocyanin content of strawberry fruits

Total anthocyanins contents of strawberry fruits were measured according to an earlier study [13], and then they were calculated using the following formula: [A530−(0.25×A657)]/M. The absorbance was measured at 530 and 657 nm by Molecular Devices (spectraMax i3X), where A530 and A657 are the absorbance at the indicated wavelengths, and M is the weight of the plant material used for extraction. All samples were measured as triplicates in three independent biological replicates.

### HPLC analysis of soluble sugars in strawberry fruits

HPLC analysis was conducted according to the previous study [13], with small modifications. In brief, it was performed with the following components and parameters: an Agilent Technologies 1,200 Series, 4.6- × 250 mm Agilent Zorbax NH2 column (Agilent technologies, CA, USA); ultrapure water as a mobile phase at a flow rate of 1.0 mL/min; a column temperature of 35°C; a refractive index detector temperature of 50°C; and an injection volume of 25 mL. The standard samples used were D-(+)Glc, D-(+)Suc (Dr Ehrenstorfer, Augsburg, Germany) and D-(–)Fru (Shanghai yuanye Bio-Technology Co., Ltd., Shanghai, China). Each sample was measured with three biological replicates.

### Transient expression assay in *Nicotiana benthamiana*

Agrobacterium-mediated transient expression assays in *N*. *benthamiana* plants were performed as previous studies [25]. All constructs were transformed into *Agrobacterium tumefaciens* strain GV3101, and *Agrobacterium* cultures were infiltrated into approximately 3-week-old leaves of *N*. *benthamiana*. Transfected *N*. *benthamiana* leaves were grown at 25 degree for 2 days before protein extraction. Immunochemical detection of GFP and imaging were carried out according to the earlier study [26], and western blot signals were quantified with ImageJ.

### Fluorescence *in situ* hybridization

The horizontal cortex region sections of strawberry fruits at Fv1 and Fv2 were produced by applying a cryostat microtome (Thermo Cryotome FSE, Thermo Fisher Scientific, US) as described previously [27]. Then fluorescence *in situ* hybridization (FISH) assay was performed according to the previous study [28]. Cortex region was incubated in hybridization buffer containing 1 μmol/ml fluorescent dye cy5-labelled oligonucleotide probes (Sangon Biotech, Co., Ltd., Shanghai, China), targeting the miR397, *FRILAIR* and *LAC11a* sequence, respectively. The fluorescent signals of detected samples were observed with a Leica TCS SP8X confocal microscope. Details of probes are listed in S9 Table.

## Results

### Genome-wide identification of lncRNAs in fruits of *F*. *vesca*

To identify lncRNAs associated with strawberry fruit ripening, we performed transcriptome sequencing on fruits of the diploid strawberry *F*. *vesca* (Hawaii 4) at three developmental stages. We generated rRNA-depleted and poly(A)-depleted libraries from strawberry samples including immature fruits with green achenes (Fv1), mature fruits with yellow achenes (Fv2), and mature fruits with brown achenes (Fv3) (S1A Fig). Each library was sequenced on the Illumina Hiseq 2500 platform, and at least 150 million paired-end reads (read length = 150 bp) were produced per library, yielding a total of ~744 million reads (S1 Table).

A modified comprehensive pipeline was employed to identify lncRNAs of *F*. *vesca* fruits, and it consisted of three main sections [29] (S1B Fig). First, reads generated from three developmental stages of fruits were mapped to the woodland strawberry reference genome (FvH4) [30,31] using RNA-STAR and then assembled into long transcripts with Scallop [32]. Second, only transcripts longer than 200 nucleotides and not overlapping annotated genes were kept, and we classified those transcripts into three types: intergenic transcripts, intronic transcripts and antisense transcripts with respect to the genomic coordinates of protein-coding genes. Third, we removed transcripts from potentially novel coding peptides/proteins through sequence similarity searches against SWISS_PROT and filtered out transcripts with internal open reading frames (ORFs) longer than 100 amino acids (aa) or ORFs longer than 50 aa at their end(s). After removing the low-expressed lncRNAs, 11,959 intergenic lncRNAs (LINC), 256 intronic lncRNAs (INTRONIC) and 13,398 antisense lncRNAs were identified (S1B Fig). Moreover, the antisense lncRNAs could be divided into two groups based on their genomic locations relative to overlapping genes: 12,706 exonic antisense lncRNAs (exonAS) and 692 intronic antisense lncRNAs (intronAS) (S2 Table).

### Characterization of lncRNAs in *F*. *vesca*

We calculated correlation coefficients of expression profiles of lncRNAs between different samples. An extremely high correlation was observed between biological replicates for each sample, but correlations between samples from different stages such as Fv1 vs Fv2, Fv1 vs Fv3 were relatively low (S2A Fig). Nevertheless, correlations between samples from different types of sequencing libraries at the same growth stage such as Fv1_polyA_Rep1 vs Fv1_rRNA_Rep1, Fv3_polyA_Rep1 vs Fv3_rRNA_Rep1 were moderately high (S2A Fig). The distribution of lncRNAs in the strawberry genome was analysed and we found that lncRNAs were widely expressed across all chromosomes (S2B Fig), which is consistent with our earlier finding in Arabidopsis [29].

We then investigated the general characteristics of lncRNAs identified in *F*. *vesca* fruits. The majority of lncRNAs identified in *F*. *vesca* were exonAS and LINC, whereas intronAS and INTRONIC were in the minority (S2 Table). Length distribution patterns were similar between exonAS and LINC, and most transcripts from both types were less than 5,000 bp, compared to the other two types of lncRNAs that were mostly less than 2,500 bp (S2C Fig). The majority of lncRNAs had only one exon (S2D Fig). With respect to GC content, there was a clear peak ranging from 30% to 50% in lncRNAs including exonAS and LINC, and a small peak with a similar distribution pattern was also observed in the other two kinds of lncRNAs (S2E Fig). To further investigate these features of lncRNAs, we compared lncRNA loci and protein-coding genes. There were statistical significant differences between lncRNAs (LINC, intronAS, exonAS and INTRONIC) and protein-coding genes in terms of transcript length (S2F Fig), number of exons (S2G Fig) and GC content (S2H Fig) (*P*-value < 0.05, Wilcoxon Rank Sum test) (S3 Table), suggesting that these general characteristics can be helpful to distinguish lncRNAs from protein-coding genes in strawberry.

Because some lncRNAs might have been involved in gene regulation *in cis*, we plotted the distance distribution between intergenic lncRNAs and their neighbour genes. Our findings suggest that intergenic lncRNAs are mainly transcribed in regions close to genes in strawberry fruits, and this trend does not have a bias with respect to the transcription direction of intergenic lncRNAs compared to their adjacent genes (S2I Fig). Furthermore, we found that some lncRNAs exhibited varied expression patterns during the maturation of strawberry fruits. Overall, plenty of lncRNAs were specifically expressed at one specific developmental stage, and a number of lncRNAs from three types of lncRNAs were constitutively expressed during the fruit ripening process (S2J Fig and S4 Table). This observation is also consistent with a previous study where *F*. *vesca* lncRNAs exhibited temporal patterns of expression in early developmental stages of strawberry fruits [7].

### Co-expression networks of lncRNAs and genes in strawberry fruits

To explore regulatory networks of lncRNAs in strawberry fruit ripening, we rebuilt co-expression networks using strawberry reference genes and lncRNAs identified here. RNA-seq datasets of 62 samples generated from previous studies [14,33,34] together with our rRNA-depleted RNA-seq data of nine samples were used in this analysis (S5 Table), and 39 co-expressed modules (S3 Fig) that represent clusters of transcripts with highly correlated expression pattern across 71 strawberry samples were reconstructed. There were between 1 and 147 lncRNAs coexpressed with reference genes in different co-expression modules (S6 Table).

Interestingly, we found that transcripts in the module “darkolivegreen4”, containing 9 lncRNAs and 124 genes, were under-expressed in fruits at unripened stages and over-expressed in fruits at ripening stages (S4A Fig). Moreover, visualization of this co-expression module showed that lncRNAs may play important roles in fruit ripening, and genes including FvH4_1g28630, FvH4_1g04670, FvH4_2g16920, FvH4_1g12320 and FvH4_3g09710 were identified as potential co-expression hubs (S4B Fig). The GO over-representation analysis of 124 genes in this co-expression module indicated that “tRNA processing”-, “intracellular protein transport”- and “protein targeting to vacuole”-related biological process terms were significantly over-represented in these genes (S4C Fig). With respect to the molecular function category, genes were significantly enriched for terms “protein binding”, “hydrolase activity” and “acetate-CoA ligase activity” (S4C Fig). The cellular component category enriched among “nuclear pore”, “membrane coat” and “NURF complex” (S4C Fig). Taken together, co-expression network results not only represent the regulatory network of lncRNAs in strawberry fruit ripening process, but also are helpful for future study of lncRNA function in strawberry fruit.

### Transcription profiling of lncRNAs in *F*. *vesca*

**Fig 1:**
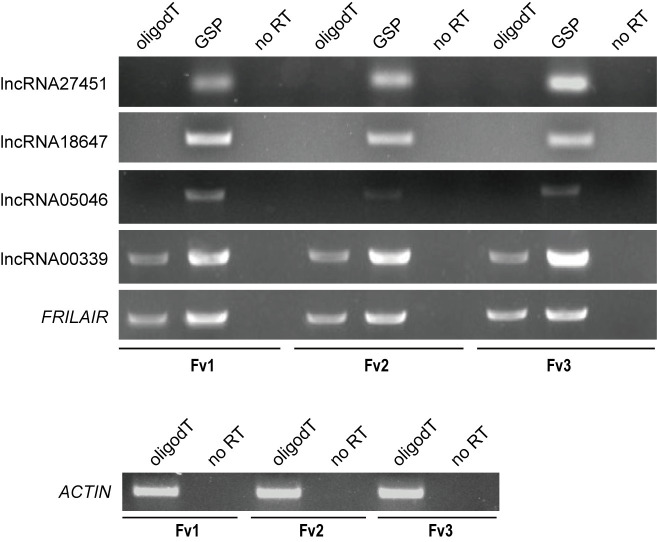
Detection of lncRNAs in strawberry fruits at three developmental stages. RT-PCR analyses were carried out on selected lncRNAs (lncRNA27451, lncRNA18647, lncRNA5046, lncRNA00339 and *FRILAIR*) in strawberry fruits from three different growth stages including Fv1, Fv2 and Fv3. Either oligo-dT or gene-specific primers (GSP) were used in the cDNA synthesis and no Reverse Transcriptase was used as negative control (no RT). Control RT-PCR using *ACTIN* primers is present on the bottom panel.

Next we used reverse transcription (RT)-PCR to validate the results from RNA-seq analysis. We chose five lncRNAs as candidates based on their polyadenylation status, including three non-polyadenylated lncRNAs (lncRNA27451, lncRNA18647 and lncRNA5046) and two polyadenylated lncRNAs (lncRNA00339 and *FRILAIR*). *FRILAIR* was chosen because it contained a miR397 target site (S7 Table) and miR397 is known to be involved in rice seed development [9] and pear fruit development [8,35]. As expected, we could not get amplicons for lncRNA27451, lncRNA18647 and lncRNA5046 using cDNA primed with oligo-dT, but amplicons were able to be detected using cDNA primed with gene-specific primers (Fig 1). LncRNA00339 and *FRILAIR* could be detected from cDNA primed with either oligo-dT or gene-specific primers (Fig 1). Subsequently, PCR amplicons of those lncRNAs were further confirmed by TA cloning, and those alignment results are consistent with our bioinformatic analysis (S5 Fig). Although five lncRNAs were expressed at all three developmental stages of strawberry fruits, their expression levels were different during the fruit ripening process (Fig 1), supporting the lncRNAs temporal expression patterns.

### *FRILAIR* acts as a noncanonical target mimic against miR397 in strawberry fruit

**Fig 2:**
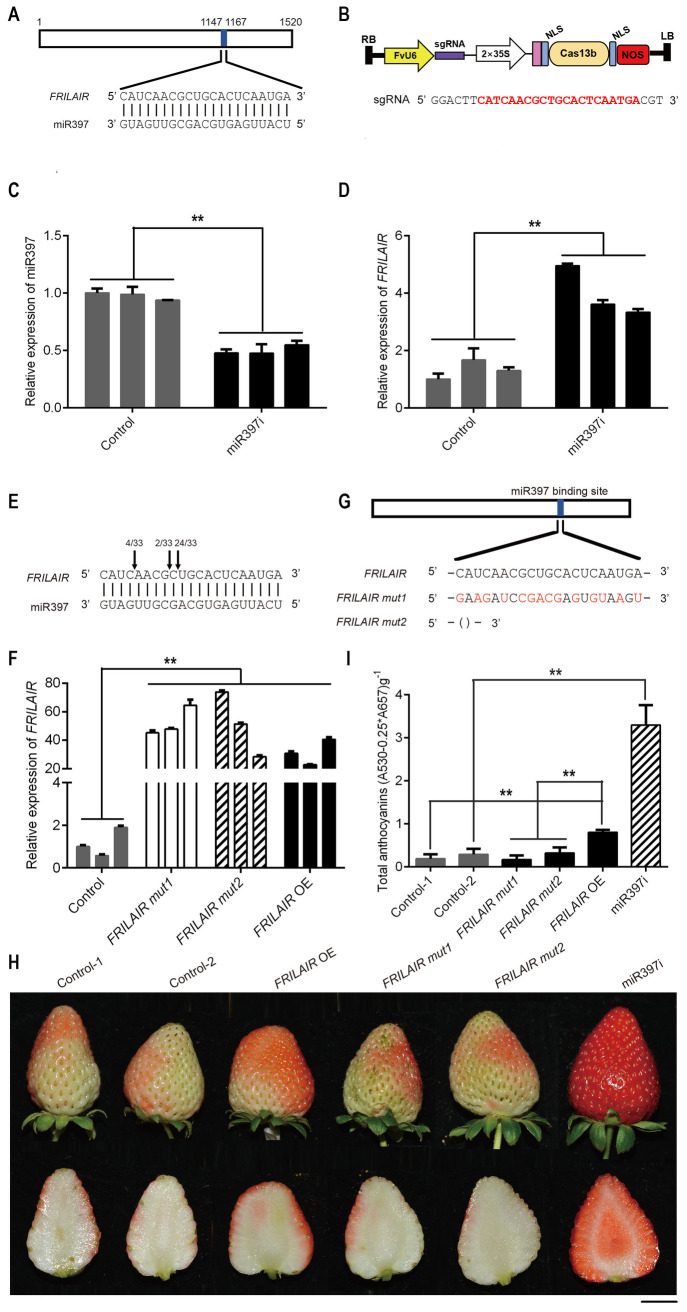
*FRILAIR* promotes strawberry ripening through working as a noncanonical target mimic of miR397. (A) Schematic diagram of *FRILAIR* showing the target site for miR397. Blue shading indicates the miR397 target site with base-pairing profile expanded below. (B) Schematic view of pFveCas13b. Protospacer sequence of sgRNA for pFveCas13b is exhibited on the bottom panel, and mature miR397 sequence is highlighted in red. (C) qRT-PCR analysis of mature miR397 expression in miR397 knockdown (miR397i) strawberry fruits. Strawberry fruits transformed with pFveCas13b vector without sgRNA were used as control. *U6* was used as the internal control. (D) qRT-PCR analysis of *FRILAIR* expression in miR397i strawberry fruits. *GAPDH* was used as the internal control. (E) miR397 cleavage sites in *FRILAIR* determined by RNA ligase-mediated 5’ RACE. Cleavage positions of *FRILAIR* are indicated by arrows, and numbers of 5’ RACE sequenced clones are shown above the arrows. (F) qRT-PCR analysis of expression of *FRILAIR* in *FRILAIR* OE, *FRILAIR mut1* and *FRILAIR mut2* over-expression fruits. Strawberry fruits transformed with empty vector were used as control. *GAPDH* was used as the internal control. (G) Schematic diagrams of *FRILAIR mut1* and *FRILAIR mut2*. (H) Phenotypic analyses of fruits from *FRILAIR* OE, *FRILAIR mut1*, *FRILAIR mut2* and miR397i. Control-1, strawberry fruits transformed with empty vector; Control-2, strawberry fruits transformed with pFveCas13b vector without sgRNA. (I) Total anthocyanin content in fruits from Control-1, Control-2, *FRILAIR* OE, *FRILAIR mut1*, *FRILAIR mut2* and miR397i. *Agrobacterium tumefaciens*-mediated transient transformations were performed on immature Falandi fruits at the big green stage. All analyses were conducted five days after infection. Statistically significant differences from control were determined by Student’s t-test: *P <0.05; **P <0.01. Values are means ±SEM of three biological replicates. Scale bar: 1 cm.

LncRNAs can bind miRNAs to repress the targeting of mRNA, thus regulating gene expression [26,36]. *FRILAIR*, a 1,520 nucleotide intergenic lncRNA found primarily in the cytoplasm (S6 and S7 Figs), was predicted to contain the miR397 binding site (Fig 2A), and miR397 has been known to be involved in rice seed development [9] and pear fruit development [8,35]. In addition, by comparing the genomic coordinates of *FRILAIR* and those for hairpin sequences of all *F*. *vesca* miRNAs annotated in miRbase, no annotated small RNAs were found by the *FRILAIR* locus.

We therefore hypothesized that *FRILAIR* could act as a binding competitor of miR397, thereby actively competing for binding with protein-coding genes targeted by miR397. First, we examined the expression levels of miR397 and *FRILAIR* in strawberry fruits at three developmental stages. *FRILAIR* expression presented a gradual increase from Fv1 to Fv3, while miR397 exhibited an opposite expression trend (S8 Fig). After that, we investigated the effect of miR397 on strawberry fruit ripening. The CRISPR/Cas13b system was employed to knock down miR397 expression in immature fruits of the octoploid strawberry Falandi, a common strawberry cultivar. CRISPR/Cas13b is a single-component programmable RNA guided RNA-targeting RNase that has both RNA processing and RNA cleaving activities [18]. A recombinant protein consisting of a 3× FLAG tag, a nuclear localization signal (NLS) and Cas13b was constructed, and this recombinant protein was expressed under the control of the 2× 35S promoter. An *F*. *vesca U6-2* promoter [19] was used to express a sgRNA targeting miR397, leading to the vector pFveCas13b-miR397 (Fig 2B). Mature miR397 expression was significantly decreased in fruits five days after infection (Fig 2C), and a remarkable increase in red colour of the cortex region and central pith were observed in miR397i fruits compared to control fruits that were transformed with pFveCas13b vector without sgRNA (Fig 2H). To characterize this phenotype quantitatively, the total anthocyanin content of fruits from miR397i and its corresponding controls were measured. Consistent with the redder phenotype, the anthocyanin content of miR397i fruits were significantly higher than controls (Fig 2I). Moreover, we over-expressed miR397 in strawberry fruits, and delayed fruit maturation was observed in miR397 OE fruits (S9A and S9B Fig). The anthocyanin content of miR397 OE fruits were significantly lower than controls (S9C Fig), which presents an opposite effect of miR397i. These data indicate that miR397 could regulate strawberry fruit ripening.

In addition, the expression level of the *FRILAIR* homolog in octoploid strawberry (S10 Fig) was significantly increased in miR397i fruits (Fig 2D). We then tested whether *FRILAIR* could be cleaved in a miR397-dependent way in strawberry fruit. RLM-RACE analysis showed that *FRILAIR* was cleaved at the position between the 12th and 13th nucleotide of miR397 in *FRILAIR* OE strawberry fruits (Fig 2E). Moreover, we checked the effect of *FRILAIR* OE on strawberry fruit ripening. *FRILAIR* expression was found to be at least eleven times higher in *FRILAIR* OE fruits compared to control fruits that were transformed with empty vector five days after infection (Fig 2F), and there was no significant difference in mi397 expression between control and *FRILAIR* OE fruits (*P* > 0.05, Student’s t-test) (S11A Fig). The cortex region of *FRILAIR* OE fruits was redder than controls (Fig 2H), which is consistent with the significantly higher total anthocyanin content of *FRILAIR* OE fruits compared to controls (Fig 2I). *FRILAIR* silencing was also performed, and delayed fruit maturation was found in *FRILAIR* KD fruits (S12A and S12D and S11C Figs). Anthocyanin content of *FRILAIR* KD fruits was significantly lower than in controls (S12C Fig), which is the reverse of *FRILAIR* OE. These observations support an important role for *FRILAIR* in strawberry fruit ripening.

Expression level of the FvH4_3g03750 and FvH4_3g03760 were not significantly change in *FRILAIR* KD fruits comparing with control (*P* > 0.05, Student’s t-test) (S6 and S13 Figs), suggesting that *FRILAIR* does not affect its neighbouring genes expressions. To further confirm the role of *FRILAIR* as a competitor for miR397 binding, two over-expression vectors containing *FRILAIR mut1* and *FRILAIR mut2* with different sequence mutations were constructed. For *FRILAIR mut1*, several nucleotide changes were introduced into the miR397 target site of *FRILAIR*, and *FRILAIR mut2* had a 21 bp deletion spanning the miR397 target site sequence (Fig 2G). The fruits over expressing *FRILAIR mut1* or *FRILAIR mut2* (Fig 2F) displayed no obvious difference in total anthocyanin content compared to controls in ripening status (Fig 2H and 2I), confirming that *FRILAIR* is cleaved in a miR397-dependent manner in strawberry fruits.

Next, we performed pairwise sequence alignments of *FRILAIR* between *F*. *vesca*, *F*. *× ananassa*, *F*. *iinumae*, *F*. *nipponica*, and *F*. *orientalis* which showed that the miR397 target site of *FRILAIR* is conserved in all of these species of *Fragaria* (S14 Fig), suggesting that the regulation of the fruit ripening process mediated by the *FRILAIR*-miR397 module is conserved across strawberry species.

### MiR397 represses strawberry fruit ripening by targeting *LAC11a*

**Fig 3:**
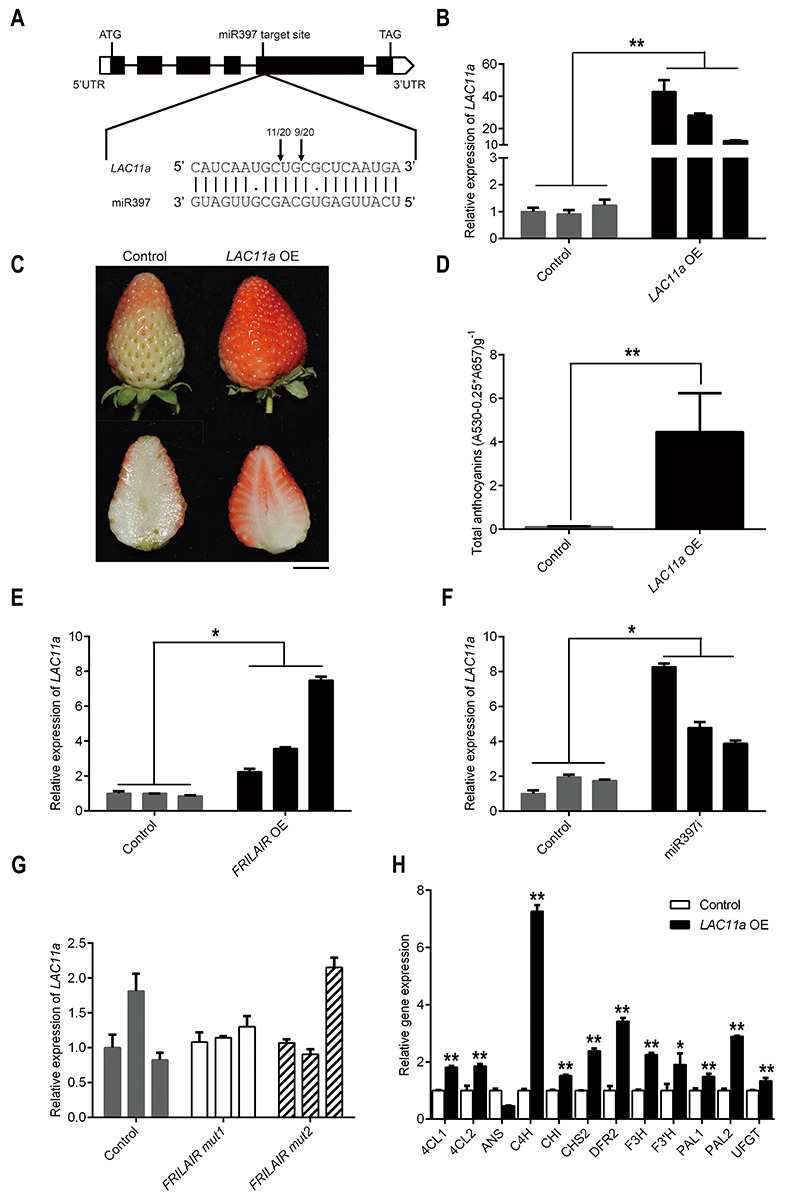
MiR397 represses strawberry fruit ripening by targeting *LAC11a*. (A) Schematic diagram of *LAC11a* presenting the target site of miR397. Boxes indicate exons (black) and untranslated regions (white, UTR) separately; black lines indicate introns. Positions of cleaved *LAC11a* are indicated by arrows, and numbers of 5’ RACE sequenced clones are shown above the arrows. (B) qRT-PCR analysis of *LAC11a* expression in *LAC11a* OE strawberry fruits. *Agrobacterium tumefaciens*-mediated transient transformations were performed on immature Falandi fruits at the big green stage, and strawberry fruits transformed with empty vector were used as control. (C) Phenotypic analysis of *LAC11a* OE fruits. Scale bar: 1 cm. (D) Total anthocyanin content of fruits from control and *LAC11a* OE. (E) qRT-PCR analysis of *LAC11a* expression in *FRILAIR* OE strawberry fruits. (F) qRT-PCR analysis of *LAC11a* expression in miR397i strawberry fruits. (G) qRT-PCR analysis of *LAC11a* on *FRILAIR mut1* and *FRILAIR mut2* over-expression fruits. (H) Relative expression levels of anthocyanin biosynthesis-related genes in fruits of *LAC11a* OE and control. All analyses were conducted five days after infection. *GAPDH* was used as the internal control. Statistically significant differences from control were determined by Student’s t-test: *P <0.05; **P <0.01. Values are means ±SEM of three biological replicates.

To further understand the regulatory network of miR397 in strawberry fruit ripening, we explored the potential protein-coding gene(s) targeted by miR397 in *F*. *vesca*. *FvH4_2g08670*, which encodes a putative laccase-11-like protein, was predicted to be one of twelve miR397 targets (S8 Table). It has 99.64% sequence similarity to the gene annotated as “maker-Fvb2-2-augustus-gene-245.25-mRNA-1” in the octoploid strawberry database [37], and we subsequently refer to “maker-Fvb2-2-augustus-gene-245.25-mRNA-1” as *LAC11a* in this study. RLM-RACE analysis showed that the mRNA of *LAC11a* was cleaved at the position between 10th and 11th nucleotide, 12th and 13th nucleotide from the 5’ end of miR397, respectively (Fig 3A), which is also consistent with an earlier study based on degradome sequencing showing that miR397 cleaves the *LAC11a* transcripts [38]. In addition, *LAC11a* expression presented a gradual increase from Fv1 to Fv3 (S15 Fig), which is similar to the *FRILAIR* expression pattern. Next, the effect of *LAC11a* on strawberry fruit maturation was investigated by over-expression. *LAC11a* OE fruits had significantly increased levels of *LAC11a* transcript but no significant change in miR397 expression (Figs 3B and S11B), and exhibited the increased fruit ripening phenotype compared to control fruits (Fig 3C). Measurements of total anthocyanin content showed that *LAC11a* OE fruit anthocyanin content was remarkably higher than control (Fig 3D). Moreover, an opposite effect was observed in *LAC11a* KD fruits (S12B–S12D and S11C Figs), further supporting a role for *LAC11a* in the regulation of fruit ripening. In addition, the mRNA levels of *LAC11a* were significantly increased in both *FRILAIR* OE and miR397i fruits (Fig 3E and 3F). However, there was no significant difference of *LAC11a* expression level between control and over-expression fruits of both *FRILAIR mut1* and *FRILAIR mut2* (Fig 3G).

**Fig 4:**
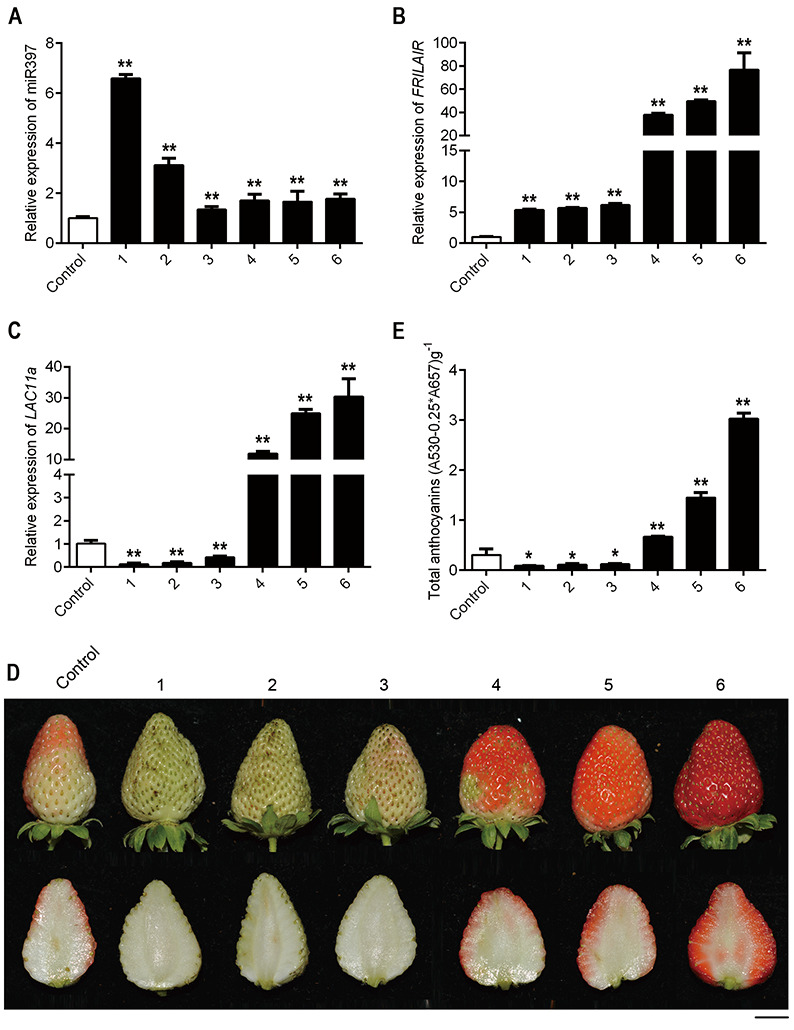
Transient co-expression of *FRILAIR* and miR397 in strawberry fruits. (A) qRT-PCR analysis of mature miR397 expression in strawberry fruits. *U6* was used as the internal control. (B) qRT-PCR analysis of *FRILAIR* expression in strawberry fruits. *GAPDH* was used as the internal control. (C) qRT-PCR analysis of endogenous *LAC11a* expression in strawberry fruits. *GAPDH* was used as the internal control. (D) Phenotypic analyses of strawberry fruits. *Agrobacterium tumefaciens*-mediated transient transformations were performed on immature Falandi fruits at the big green stage. (E) Measurement on total anthocyanin contents of strawberry fruits. Strawberry fruits were co-transformed with both *FRILAIR* and miR397 over-expressing vectors. 1–3: strawberry fruits co-expressing OD600 = 0.4 miR397 OE and OD600 = 0.4 *FRILAIR* OE; 4–6: strawberry fruits coexpressing OD600 = 0.4 miR397 OE and OD600 = 1.0 *FRILAIR* OE. All analyses were conducted five days after infection, and strawberry fruits transformed with empty vector were used as control. Statistically significant differences from control were determined by Student’s t-test: *P <0.05; **P <0.01. Values are means ±SEM of three biological replicates. Scale bar: 1 cm.

To further investigate the expression correlation between miR397, *FRILAIR* and *LAC11a* during the strawberry fruit ripening process, we transiently co-expressed *FRILAIR* and miR397 at different concentration combinations in strawberry fruits and examined endogenous *LAC11a* expression levels in those fruits. The expression of *LAC11a* was strikingly reduced in fruits with a higher ratio of miR397 to *FRILAIR*, and an opposite expression trend of *LAC11a* was present in fruits with a lower ratio of miR397 to *FRILAIR* (Fig 4A–4C). Consistent with the expression trend of *LAC11a*, delayed fruit maturation was observed in fruits where *LAC11a* was down-regulated and accelerated fruit maturation was detected in fruits where *LAC11a* was up-regulated (Fig 4D). Moreover, these observations were supported by total anthocyanin content measurement (Fig 4E), further demonstrating that there is an interplay among *FRILAIR*, miR397 and *LAC11a* in the strawberry ripening process. Although both *FRILAIR* and *LAC11a* could be cleaved by miR397, the predicted ΔG of binding [39] of the miR397 with *FRILAIR* is lower than that with its target *LAC11a* (S16 Fig), indicating that either of them can form equally stable RNA duplexes with miR397. This result is also in line with the decoy mechanism found in human [40]. In addition, the effect of *FRILAIR* on miR397-dependent *LAC11a* accumulation was investigated with transient expression assays in *Nicotiana benthamiana*. First, *FRILAIR*, miR397 and a *LAC11a*:*GFP* reporter were transiently expressed in *N*. *benthamiana* (S17B and S18A–S18F Figs). As expected, LAC11a:GFP protein accumulation was reduced after overexpression of miR397 (S17C Fig), and the effect of miR397 on *LAC11a*:*GFP* was suppressed by simultaneous expression of *FRILAIR* (S17C Fig). These results are consistent with the finding in strawberry fruits (Fig 4), which also indicates that the transient expression assays in *N*. *benthamiana* is an effective method to study the noncanonical target mimicry mediated by *FRILAIR* in strawberry. In addition, to prove that *FRILAIR* is a direct target of miR397, we generated constructs including *GFP*:*FRILAIR*, *GFP*:*FRILAIRmut2* and *GFP*:*FRILAIRmut3* (S19A and S19B Fig), and these constructs were co-expressed with miR397 in *N*. *benthamiana* (S19C Fig). *GFP*:*FRILAIR* protein accumulation was decreased after over-expression of miR397 compared with expression of either *GFP*:*FRILAIRmut2* or *GFP*:*FRILAIRmut3* (S19D Fig), which is in accordance with our expectation. Then, two miR397 mimic constructs including Fmimic1 and Fmimic2 were generated according to an earlier study [41]. A three-nucleotide bulge was added between 10–11 bp and 12–13 bp in the miR397 target site of *FRILAIR* for Fmimic1 and Fmimic2, respectively (S17A Fig). We found that LAC11a:GFP protein accumulation was remarkably reduced after co-expression of both miR397 and *FRILAIR mut2* compared with co-expression of both miR397 and *FRILAIR* (S17D and S18G–S18L Figs). Moreover, compared with co-expression of both miR397 and *FRILAIR*, the amount of LAC11a:GFP protein was significantly higher when Fmimic2 and miR397 were simultaneous expressed (*P* < 0.05, Student’s t-test), but the difference of LAC11a:GFP protein level was not significant between coexpression miR397 and *FRILAIR* and co-expression miR397 and Fmimic1 (*P* > 0.05, Student’s t-test). This is possibly because that the cleavage position of *FRILAIR* is between the 12th and 13th nucleotide of miR397, and these results together further support that *FRILAIR* functions as a noncanonical target mimic.

Finally, we explored the underlying mechanism for anthocyanin content increase in *LAC11a* OE fruits, via the expression of genes involved in anthocyanin biosynthesis [13,42]. We found that the majority of those genes including *4CL1*, *4CL2*, *C4H*, *CHI*, *CHS2*, *DFR2*, *F3H*, *F3’H*, *PAL1*, *PAL2* and *UFGT* were up-regulated in *LAC11a* OE fruits (Fig 3H), indicating that *LAC11a* promotes anthocyanin synthesis. Moreover, we explored these genes’ expression in *FRILAIR* OE and miR397i fruits, and found that most of them were up-regulated in *FRILAIR* OE fruits (S20A Fig) and miR397i fruits (S20B Fig), indicating that the *FRILAIR*-miR397 module can promote anthocyanin synthesis in strawberry fruits. Taken together, our results indicate that *FRILAIR* has a regulatory role in fruit ripening as a noncanonical target mimic for miR397, by de-repressing *LAC11a* expression.

### *FRILAIR* regulates sugar accumulation in strawberry fruit

**Fig 5:**
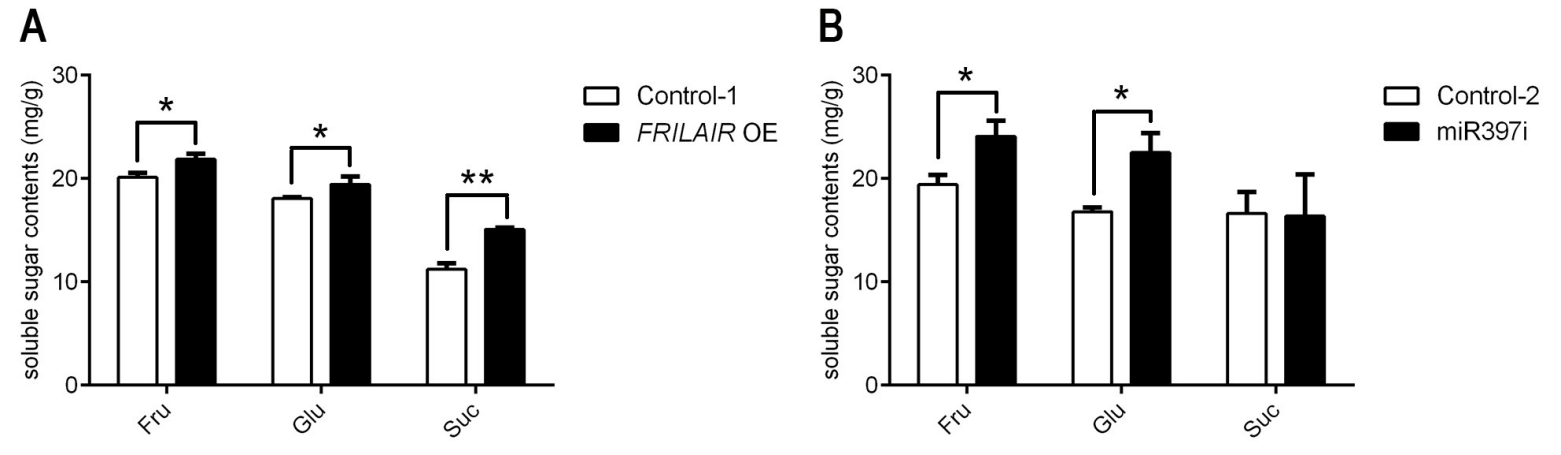
Changes in soluble sugar content of Suc, Glu and Fru in strawberry fruits. (A) Soluble sugar content of *FRILAIR* OE fruits. (B) Soluble sugar contents of miR397i fruits. Statistically significant differences from control were determined by Student’s t-test: *P <0.05; **P <0.01. Values are means ±SEM of three biological replicates.

For fruit maturation, colour is only one important indicator of quality and is very helpful for the judgment of fruit ripeness. Nonetheless, there are a series of physiological, biochemical, and organoleptic changes besides colour involved in fruit ripening, such as maintenance of cell wall structure and sugar accumulation. In *Populus*, *LAC2* targeted by miR397a is involved in sugar release [43,44], suggesting that miR397 targets other than *LAC11a* might be involved in other strawberry ripening associated traits. The main soluble sugars that accumulate in strawberry fruits were measured with HPLC, including sucrose, glucose and fructose [15]. The amounts of two soluble sugars containing glucose and fructose were significantly increased in both *FRILAIR* OE and miR397i fruits comparing with controls (Fig 5). Sucrose content was significantly higher in *FRILAIR* OE fruits compared to controls, but there was not a significant difference between miR397i fruits and controls (Fig 5). This observation makes sense in the context of an earlier finding that sucrose levels decline after an initial increase during the strawberry fruit ripening process [45]. This is possibly because sucrose is converted to glucose and fructose by invertase, resulting in an increase of glucose and fructose and a decrease in sucrose content [45,46]. These results also support the finding that fruits of miR397i were more mature than *FRILAIR* OE fruits based on total anthocyanin content.

## Discussion

It has been known that lncRNAs play important roles in diverse biological processes in plants. In strawberry, thousands of lncRNAs have been identified in flower and fruit tissues based on RNA-seq datasets [7]. However, their biological functions in fruit ripening are still unknown. In this study, we have identified a surprisingly large number (25,613) of lncRNAs by combining rRNA-depleted RNA sequencing with poly(A)-depleted RNA sequencing in strawberry. A set of these lncRNAs exhibited temporal expression specificity, implying specific roles for lncRNAs at multiple stages of strawberry fruit development.

**Fig 6:**
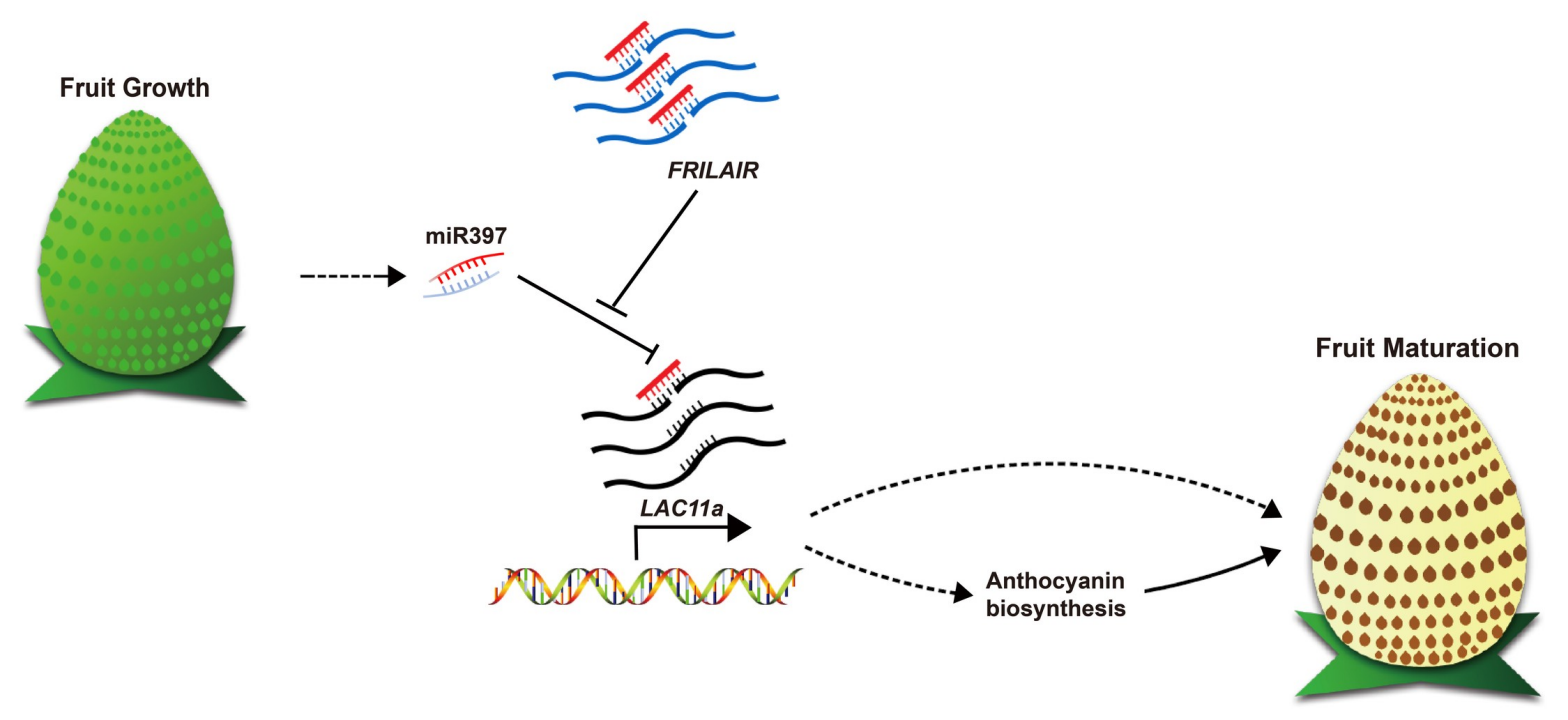
A proposed model illustrating the endogenous competitiveness between *FRILAIR*, miR397 and *LAC11a* in modulating strawberry fruit ripening. Both *FRILAIR* and *LAC11a* mRNAs can be cleaved by miR397. Accumulation of *FRILAIR* releases the *LAC11a* repression, which subsequently promotes expressions of genes involved in anthocyanin biosynthesis pathway, leading to the acceleration of strawberry fruit ripening. Solid lines represent regulatory links observed in strawberry, and dashed lines represent upstream and downstream elements of the gene regulatory network for fruit ripening. Arrows indicate positive regulation, and blunt-ended bars indicate inhibition.

LncRNAs containing binding sites of miRNAs could act as a noncanonical target mimic and there are experimental examples in mammals supporting this supposition, which is named as the competing endogenous RNA (ceRNA) [40]. Our results support a model wherein the lncRNA *FRILAIR* functions as a noncanonical target mimic that can bind to miR397 in strawberry fruit, thus alteration of *FRILAIR* abundance can modulate the activity of miR397 on its downstream protein-coding genes. Consistent with this proposed model, over-expression of *FRILAIR* results in the increase of *LAC11a* transcriptional levels in strawberry fruits, and this trend is also observed in miR397i fruits. Moreover, we found that miR397 represses strawberry fruit ripening by targeting *LAC11a*, whereas accumulation of *FRILAIR* transcripts could release this repression, leading to the promotion of strawberry fruit ripening (Fig 6). In addition, miR397 expressed highly on cortex region of immature strawberry fruits, but both *FRILAIR* and *LAC11a* presented low expression levels (S21 Fig). Nevertheless, in mature fruits, miR397, *FRILAIR* and *LAC11a* exhibited the opposite expression trend (S22 Fig). Here, our findings suggest a role for miR397 in repressing strawberry fruit ripening by targeting *LAC11a*. Interestingly, both *LAC11a* and *LAC11b* were known to be targeted by miR397 [38], however, the expression levels of *LAC11b* were extremely low in strawberry fruits at three developmental stages studied here, suggesting that *LAC11b* might play different roles in early strawberry fruit development. In addition, the red color of strawberry fruits comes from anthocyanins stored in vacuoles, and ABA has been found to affect fruit anthocyanin biosynthesis in strawberry [13]. ABA catabolism and biosynthesis also play important roles in strawberry fruit development and ripening [15,16]. In *Arabidopsis thaliana*, expression of several laccases (*LAC5*, *LAC12* and *LAC13*) were up-regulated in response to ABA [47]. Therefore, the potential correlation between *LAC11a* and ABA regulated strawberry ripening should be investigated in future.

In conclusion, we have characterized a functional model for lncRNA-miRNA-gene regulation in the regulation of strawberry fruit ripening. Our findings suggest that the endogenous competing action of lncRNAs containing binding sites for miRNAs might be a common regulatory mechanism in plants. We suggest that lncRNAs may provide a valuable resource for the manipulation of the gene regulatory network responsible for fruit ripening in the strawberry.

## Supporting information

S1 Fig Identification of lncRNAs in *F*. *vesca*. (A) Exemplars of *F*. *vesca* fruits at three developmental stages. Fv1, immature fruits with green achenes; Fv2, mature fruits with yellow achenes; Fv3, mature fruits with brown achenes. Scale bar: 1.5 mm. (B) Flowchart of lncRNA identification in strawberry.
(TIF)

S2 Fig Characterization of lncRNAs in *F*. *vesca*. (A) Correlation matrix showing the correlation of global expression profiles of lncRNAs across individual samples. “polyA” represents polyA-depleted libraries, and “rRNA” represents rRNA-depleted libraries. Pairwise correlation was calculated based on expression values of all lncRNAs using Pearson correlation. Samples named with “Rep1~3” represent three biological replicates. (B) Genomic distribution of lncRNAs and genes. For each chromosome, the density plot in the top panel (coloured red) represents the frequency of lncRNAs in each 100 kb genomic bin; the heatmap in the bottom panel (coloured blue) represents the frequency of protein-coding genes in each 100 kb genomic bin. (C) Length distribution of lncRNAs identified in strawberry fruits. ExonAS: exonic antisense lncRNAs; intronAS: intronic antisense lncRNAs; INTRONIC: intronic lncRNAs; LINC: intergenic lncRNAs. (D) Exon numbers of lncRNAs identified in strawberry fruits. (E) GC-content percentage for lncRNAs identified in strawberry fruits. (F) Length comparison of lncRNAs and protein-coding genes. (G) Exon number comparison of lncRNAs and protein-coding genes. (H) GC content comparison of lncRNAs and protein-coding genes. (I) Distribution of lincRNA distances from neighbour genes. Values on the X axis to the left of “0” represent lincRNAs located at the 5’ end of neighbour genes, where “0” stands for the lincRNA transcription start site (TSS); values to the right of “0” represent distances of lincRNAs from the 3’ end of neighbor genes, where “0” represents the lincRNA transcription termination site (TTS). (J) Expression patterns of lncRNAs identified in strawberry fruits. Expression values of lncRNAs were normalized using the Variance-Stabilizing Transformation (VST) method based on CPM (Counts per million). LincRNAs, intronic lncRNAs and antisense lncRNAs are marked in orange, blue and green, respectively.
(TIF)

S3 Fig Co-expression networks between lncRNAs and reference genes in strawberry. (TIF)

S4 Fig Co-expression networks between lncRNAs and annotated genes in strawberry. (A) A representative co-expression module containing lncRNAs and genes. Transcripts’ expression patterns in the co-expression module/sub-network “darkolivegreen4” are presented in the top panel, and the bar-plot in the bottom panel displays the eigengene values. Red means “over-expressed” and green means “under-expressed” in the heatmap. The “eigengene value” stands for the gene expression profiles in this module, which is defined as the first principal component of the module. (B) Graphical representation of coexpression module/sub-network “darkolivegreen4”. The top 50 connections based on co-expression weights in module “darkolivegreen4”. Nodes with labels colored in blue represent lncRNAs. Node size and node label size are inversely proportional to “Average shortest path length”. The color gradient in edges is proportional to co-expression weights. (C) GO enrichment analysis for reference genes in the co-expression module “darkolivegreen4”. The top 10 most statistically significant over-represented GO terms in each category for genes in module “darkolivegreen4”. Over-representation was calculated using a hypergeometric test based on all reference genes annotated in strawberry. Statistically significant over-represented GO terms were selected based on *p*-value < 0.05.
(TIF)

S5 Fig Sequence chromatograms of lncRNAs tested by RT-PCR. (A) lncRNA27451, (B) lncRNA18647, (C) lncRNA05046, (D) lncRNA00339, (E) *FRILAIR*. Primer pairs used in RT-PCR are shown as green arrows. Amplicon stands for Sanger sequencing results from TA cloning, and more than one sequencing reactions were performed for amplicons of lncRNA00339 and *FRILAIR*.
(TIF)

S6 Fig Schematic diagram of *FRILAIR* locus in strawberry genome. Pink arrow represents *FRILAIR*. Number above each lane stand for the distance to *FRILAIR* transcription start site.
(TIF)

S7 Fig Relative subcellular (nuclear or cytoplasmic) distributions of *FRILAIR* transcripts. (A) Glutamine tRNA as the cytoplasmic RNA control (relative value set to 1 in cytoplasmic fraction), (B) *U6* RNA as nuclear RNA control (relative value set to 1 in nuclear fraction), (C) total *FRILAIR* mRNA. Nu, nuclear fraction; Ct, cytoplasmic fraction.
(TIF)

S8 Fig Expression patterns of *FRILAIR* and miR397 in *F*. *vesca*. (A) Expression pattern of *FRILAIR* in strawberry fruit at three developmental stages in *F*. *vesca*. *GAPDH* was used as the internal control. (B) Expression pattern of mature miR397 in strawberry fruit at three developmental stages in *F*. *vesca*. *U6* was used as the internal control. Statistically significant differences from control were determined by Student’s t-test: *P <0.05; **P <0.01. Values are means ±SD of three biological replicates.
(TIF)

S9 Fig Overexpression of miR397 delays fruit maturation. (A) qRT-PCR analysis of miR397 expression in miR397OE fruits. *Agrobacterium tumefaciens*-mediated transient transformations were performed on immature Falandi fruits at the big green stage, and strawberry fruits transformed with empty vector were used as control. *U6* was used as the internal control. (B) Phenotypic analyses of fruits from miR397 OE. (C) Total anthocyanin content in fruits from Control and miR397 OE. The analysis was conducted five days after infection. Statistically significant differences from control were determined by Student’s t-test: **P <0.01. Values are means ±SEM of three biological replicates. Scale bar: 1 cm.
(TIF)

S10 Fig Sequence pairwise alignment of *FRILAIR* in *F*. *vesca* and *F*. *× ananassa*. The red line indicates the miR397 target site of *FRILAIR*, and primers used in qRT-PCR are shown as black arrows.
(TIF)

S11 Fig qRT-PCR analyses of miR397 expression in strawberry. (A) Expression levels of mature miR397 in *FRILAIR* OE fruits. (B) Expression levels of mature miR397 in *LAC11a* OE fruits. (C) Expression levels of mature miR397 in *FRILAIR* KD and *LAC11a* KD fruits. *U6* was used as the internal control. Values are means ±SEM of three biological replicates.
(TIF)

S12 Fig Knockdown of *FRILAIR* and *LAC11a* delay fruit maturation. (A) qRT-PCR analysis of *FRILAIR* expression in *FRILAIR* KD fruits. Two independent sgRNAs targeted different regions of *FRILAIR* were applied to achieve two independent *FRILAIR* KD fruits including *FRILAIR* KD-1 and *FRILAIR* KD-2. (B) qRT-PCR analysis of *LAC11a* expression in *LAC11a* KD fruits. Two independent sgRNAs targeted different regions of *LAC11a* were applied to achieve two independent *LAC11a* KD fruits including *LAC11a* KD-1 and *LAC11a* KD-2. (C) Phenotypic analyses of fruits from *FRILAIR* KD and *LAC11a* KD. (D) Total anthocyanin content in fruits from Control, *FRILAIR* KD and *LAC11a* KD. Strawberry fruits transformed with pFveCas13b vector without sgRNA were used as control. *GAPDH* was used as the internal control. *Agrobacterium tumefaciens*-mediated transient transformations were performed on immature Falandi fruits at the big green stage. All analyses were conducted five days after infection. Statistically significant differences from control were determined by Student’s t-test: **P <0.01. Values are means ±SEM of three biological replicates. Scale bar: 1 cm.
(TIF)

S13 Fig qRT-PCR analysis of FvH4_3g03750 and FvH4_3g03760 expression in *FRILAIR* KD fruits. (A) Expression level of FvH4_3g03750 in *FRILAIR* KD fruits. (B) Expression level of FvH4_3g03760 in *FRILAIR* KD fruits. *GAPDH* was used as the internal control. Error bars represent SEM from three replicates.
(TIF)

S14 Fig Sequence pairwise alignment of *FRILAIR* among *Fragaria* species. Sequence pairwise alignment was performed on *FRILAIR* from *F*. *vesca*, *F*. *× ananassa*, *F*. *iinumae*, *F*. *nipponica* and *F*. *orientalis*. The red box indicates the highly conserved miR397 target site.
(TIF)

S15 Fig Expression pattern of *LAC11a* in strawberry fruit at three different developmental stages in *F*. *vesca*. Statistically significant differences from control were determined by Student’s t-test: *P <0.05; **P <0.01. Values are means ±SD of three biological replicates.
(TIF)

S16 Fig Base pairing of the miR397 with its predicted binding sites on its targets. ΔG values were obtained from miRanda (Enright *et al*., 2003).
(TIF)

S17 Fig Effect of *FRILAIR* on *LAC11a* accumulation requires base-pairing between *FRILAIR* and miR397. (A) Schematic diagrams of Fmimic1 and Fmimic2. (B) Diagrams of constructs used in the transient expression assay. (C and D) Transient expression assays in *N*. *benthamiana*, monitoring *LAC11a*:*GFP* by western blot. Relative LAC11a:GFP accumulation in the different agroinfiltration assays is indicated in bar graphs below each panel. *Tubulin* is shown as a loading control. Error bars represent SEM from three replicates.
(TIF)

S18 Fig qRT-PCR analyses of target gene expression in *N*. *benthamiana* plants. (A) Expression levels of miR397 in leaves of tobacco transiently expressed vectors including *LAC11a*:*GFP* + miR397 and *LAC11a*:*GFP* + *FRILAIR* + miR397 vectors. (B) Expression levels of *FRILAIR* in leaves of tobacco transiently expressed vectors including *LAC11a*:*GFP* + *FRILAIR* and *LAC11a*:*GFP* + *FRILAIR* + miR397 vectors. (C) Expression levels of *LAC11a* in leaves of tobacco transiently expressed vectors including *LAC11a*:*GFP*, *LAC11a*:*GFP* + *FRILAIR*, *LAC11a*:*GFP* + miR397 and *LAC11a*:*GFP* + *FRILAIR* + miR397 vectors. (D) Expression levels of *Basta* in leaves of tobacco transiently expressed vectors including *LAC11a*:*GFP* + miR397 and *LAC11a*:*GFP* + *FRILAIR* + miR397 vectors. (E) Expression levels of *NPTII* in leaves of tobacco transiently expressed vectors including *LAC11a*:*GFP* + *FRILAIR* and *LAC11a*:*GFP* + *FRILAIR* + miR397 vectors. (F) Expression levels of *HYG* in leaves of tobacco transiently expressed vectors including *LAC11a*:*GFP*, *LAC11a*:*GFP* + *FRILAIR*, *LAC11a*:*GFP* + miR397 and *LAC11a*:*GFP* + *FRILAIR* + miR397 vectors. (G-L) Expression levels of miR397, *FRILAIR*, *LAC11a*, *Basta*, *NPTII* and *HYG* in leaves of tobacco transiently expressed vectors including *LAC11a*:*GFP* + *FRILAIR* + miR397, *LAC11a*:*GFP* + Fmimic1 + miR397, *LAC11a*:*GFP* + Fmimic2 + miR397 and *LAC11a*:*GFP* + *FRILAIR mut2* + miR397 vectors, respectively. *Tubulin* was used as the internal control. Error bars represent SEM from three replicates.
(TIF)

S19 Fig *FRILAIR* is a direct target of miR397. (A) Schematic diagrams of *FRILAIR mut2* and *FRILAIR mut3*. (B) Diagrams of constructs used in the transient expression assay. (C) Expression levels of *Basta*, *HYG*, miR397 and *GFP* in tobacco leaves transiently expressing *GFP*:*FRILAIR* + miR397, *GFP*:*FRILAIR mut2* + miR397 and *GFP*:*FRILAIR mut3* + miR397 constructs. *Tubulin* was used as the internal control. *HYG* stands for the hygromycin resistance gene. (D) Transient expression assays in *N*. *benthamiana*, monitoring *GFP*:*FRILAIR* by western blot. Relative *GFP*:*FRILAIR* accumulation in the different agroinfiltration assays is indicated in bar graphs below each panel. Tubulin is shown as a loading control. Error bars represent SEM from three replicates.
(TIF)

S20 Fig **Relative expression levels of anthocyanin biosynthesis-related genes in fruits of *FRILAIR* OE (A) and miR397i (B).** All analyses were conducted five days after infection. *GAPDH* was used as the internal control. Statistically significant differences from control were determined by Student’s t-test: *P <0.05; **P <0.01. Values are means ±SEM of three biological replicates.
(TIF)

S21 Fig Spatial expression pattern analysis of miR397, *LAC11a* and *FRILAIR* in strawberry fruit at Fv1. FISH was performed on horizontal sections of the cortex region. Images of (I) were observed with a Leica TCS SP8X confocal microscope under 10× magnification, and images of (II) were enlargements of the boxed area in (I) from the respective samples. Red fluorescence indicates the presence of corresponding RNAs detected by cy5-labeled riboprobes for miR397, *LAC11a* and *FRILAIR*, respectively. Mock hybridization used a cy5-labeled riboprobe that has no identity to the strawberry genome, and cy5 fluorescence signals were not observed. C, cortex region; M, margin region. Scale bars: 250 μm in (I), 50 μm in (II).
(TIF)

S22 Fig Spatial expression pattern analysis of miR397, *LAC11a* and *FRILAIR* in strawberry fruit at Fv2. FISH was performed on horizontal sections of the cortex region. Images of (I) were observed with a Leica TCS SP8X confocal microscope under 10× magnification, and images of (II) were enlargements of the boxed area in (I) from the respective samples. C, cortex region; M, margin region. Scale bars: 250 μm in (I), 50 μm in (II).
(TIF)

S1 Table Summary of RNA-seq data used in this study. (XLSX)

S2 Table Basic statistics of identified lncRNAs in this study, including length, number of exons and GC percentage. (XLSX)

S3 Table Statistical *P*-value of lncRNA and gene in length, number of exons and GC percentage. (XLSX)

S4 Table Expression values of identified lncRNAs in 18 individual samples. Expression values were normalised as Counts Per Million reads (CPM) with Trimmed Mean of M-values (TMM) methods.
(XLSX)

S5 Table Sample lists of gene co-expression networks analysis. (XLSX)

S6 Table Summary of co-expression networks analysis. (XLSX)

S7 Table Potential miRNAs binding site in lncRNAs. (XLSX)

S8 Table Potential miR397 targets found in strawberry. (XLSX)

S9 Table Sequences of primers used in this study. (XLSX)
