# Supplementary figures and images for: The long noncoding RNA FRILAIR regulates strawberry fruit ripening by functioning as a noncanonical target mimic
Source: PLoS Genet. 2021 Mar 19;17(3):e1009461. doi: 10.1371/journal.pgen.1009461 (PMC8011760; doi:10.1371/journal.pgen.1009461)

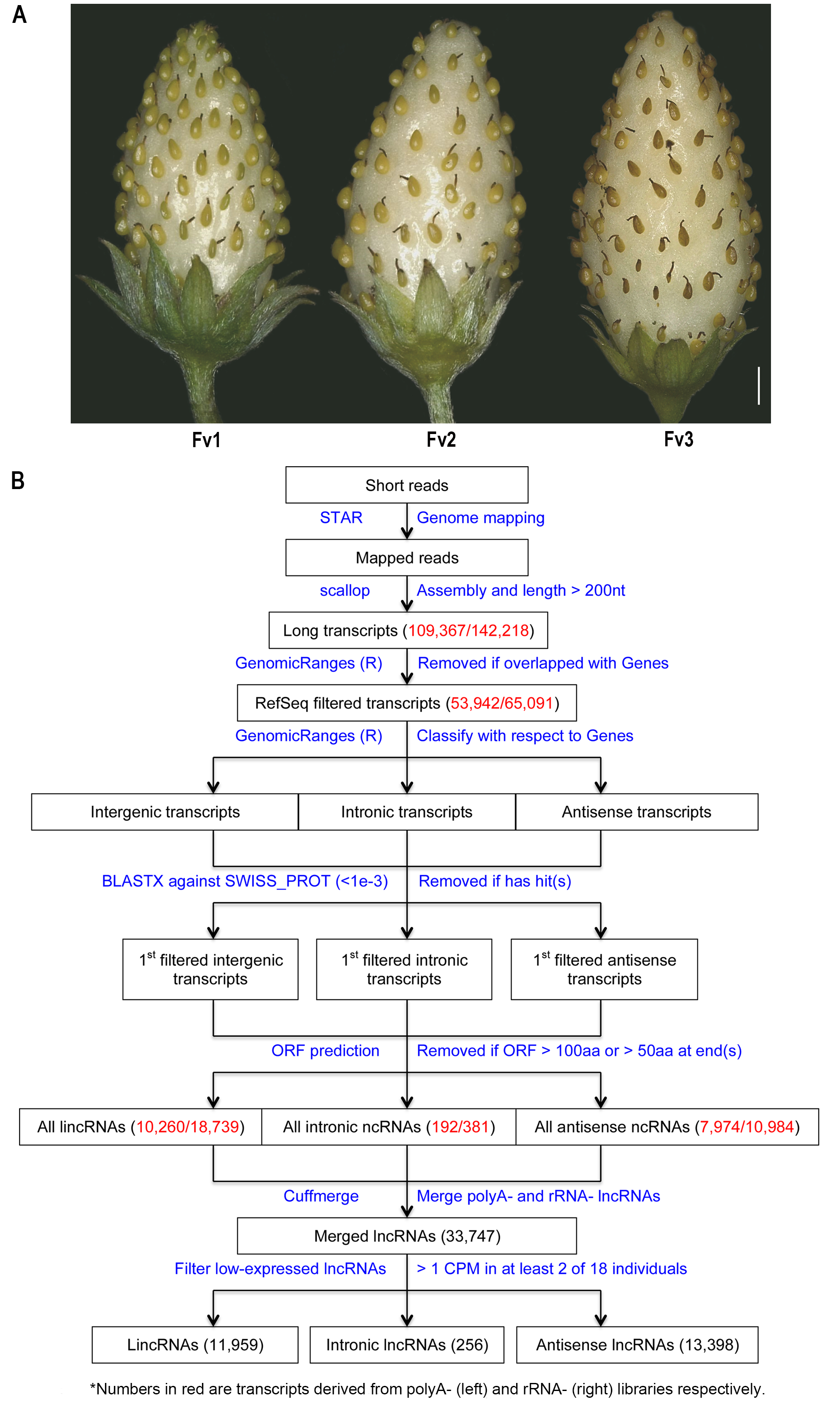

Supplement: S1 Fig — (A) Exemplars of F. vesca fruits at three developmental stages. Fv1, immature fruits with green achenes; Fv2, mature fruits with yellow achenes; Fv3, mature fruits with brown achenes. Scale bar: 1.5 mm. (B) Flowchart of lncRNA identification in strawberry. (TIF) [file pgen.1009461.s001.tif]

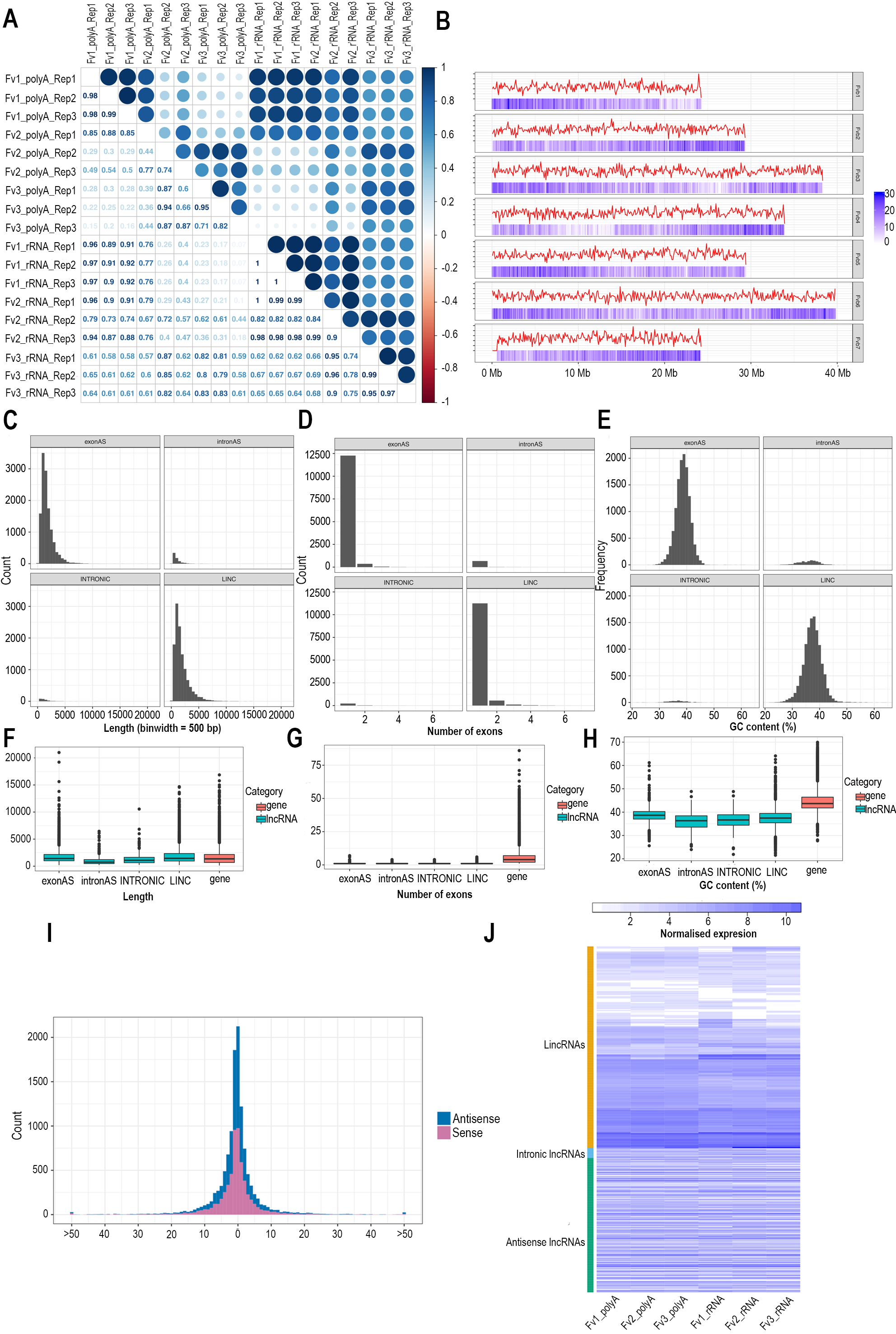

Supplement: S2 Fig — (A) Correlation matrix showing the correlation of global expression profiles of lncRNAs across individual samples. “polyA” represents polyA-depleted libraries, and “rRNA” represents rRNA-depleted libraries. Pairwise correlation was calculated based on expression values of all lncRNAs using Pearson correlation. Samples named with “Rep1~3” represent three biological replicates. (B) Genomic distribution of lncRNAs and genes. For each chromosome, the density plot in the top panel (coloured red) represents the frequency of lncRNAs in each 100 kb genomic bin; the heatmap in the bottom panel (coloured blue) represents the frequency of protein-coding genes in each 100 kb genomic bin. (C) Length distribution of lncRNAs identified in strawberry fruits. ExonAS: exonic antisense lncRNAs; intronAS: intronic antisense lncRNAs; INTRONIC: intronic lncRNAs; LINC: intergenic lncRNAs. (D) Exon numbers of lncRNAs identified in strawberry fruits. (E) GC-content percentage for lncRNAs identified in strawberry fruits. (F) Length comparison of lncRNAs and protein-coding genes. (G) Exon number comparison of lncRNAs and protein-coding genes. (H) GC content comparison of lncRNAs and protein-coding genes. (I) Distribution of lincRNA distances from neighbour genes. Values on the X axis to the left of “0” represent lincRNAs located at the 5’ end of neighbour genes, where “0” stands for the lincRNA transcription start site (TSS); values to the right of “0” represent distances of lincRNAs from the 3’ end of neighbor genes, where “0” represents the lincRNA transcription termination site (TTS). (J) Expression patterns of lncRNAs identified in strawberry fruits. Expression values of lncRNAs were normalized using the Variance-Stabilizing Transformation (VST) method based on CPM (Counts per million). LincRNAs, intronic lncRNAs and antisense lncRNAs are marked in orange, blue and green, respectively. (TIF) [file pgen.1009461.s002.tif]

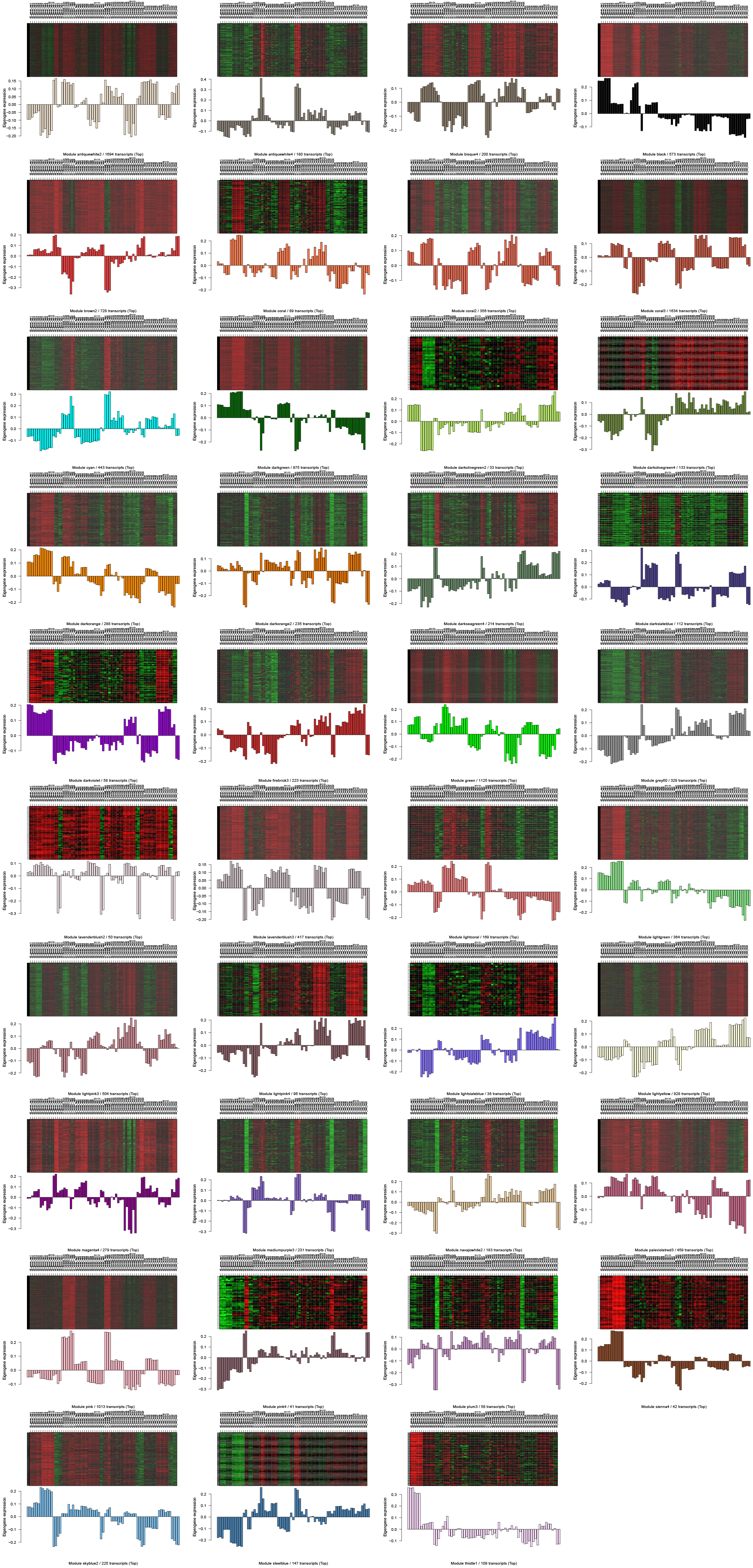

Supplement: S3 Fig — (TIF) [file pgen.1009461.s003.tif]

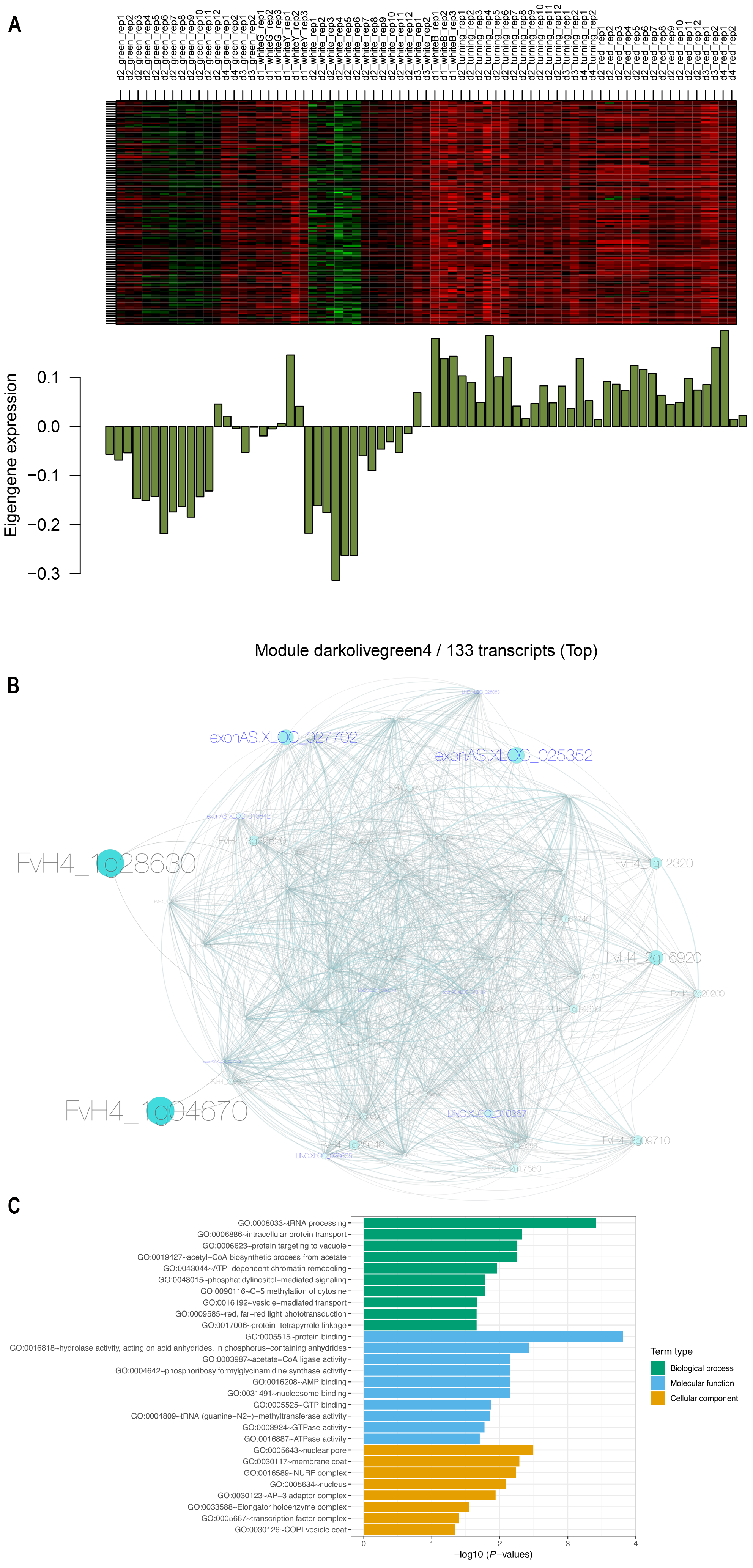

Supplement: S4 Fig — (A) A representative co-expression module containing lncRNAs and genes. Transcripts’ expression patterns in the co-expression module/sub-network “darkolivegreen4” are presented in the top panel, and the bar-plot in the bottom panel displays the eigengene values. Red means “over-expressed” and green means “under-expressed” in the heatmap. The “eigengene value” stands for the gene expression profiles in this module, which is defined as the first principal component of the module. (B) Graphical representation of coexpression module/sub-network “darkolivegreen4”. The top 50 connections based on co-expression weights in module “darkolivegreen4”. Nodes with labels colored in blue represent lncRNAs. Node size and node label size are inversely proportional to “Average shortest path length”. The color gradient in edges is proportional to co-expression weights. (C) GO enrichment analysis for reference genes in the co-expression module “darkolivegreen4”. The top 10 most statistically significant over-represented GO terms in each category for genes in module “darkolivegreen4”. Over-representation was calculated using a hypergeometric test based on all reference genes annotated in strawberry. Statistically significant over-represented GO terms were selected based on p-value < 0.05. (TIF) [file pgen.1009461.s004.tif]

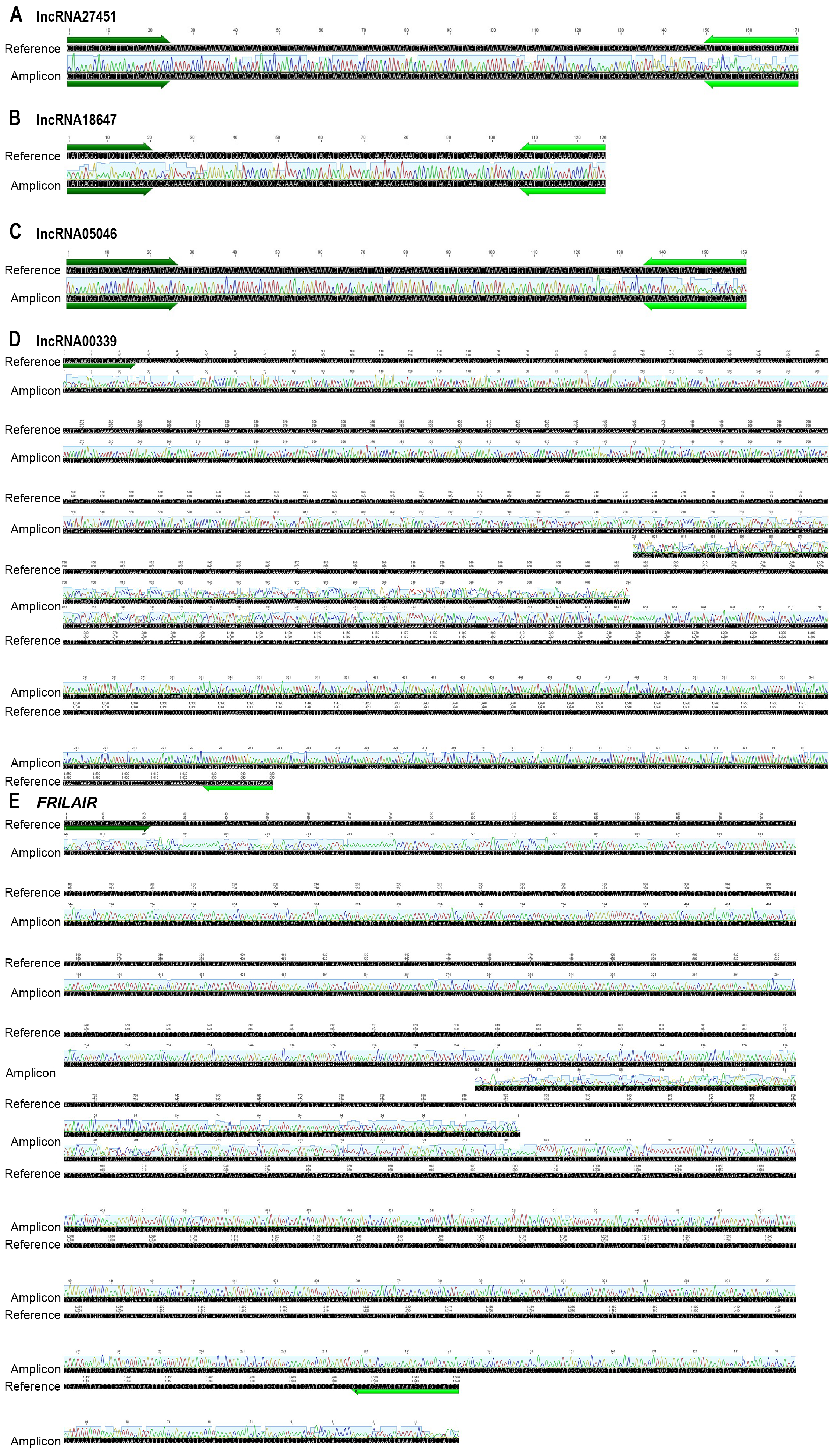

Supplement: S5 Fig — (A) lncRNA27451, (B) lncRNA18647, (C) lncRNA05046, (D) lncRNA00339, (E) FRILAIR. Primer pairs used in RT-PCR are shown as green arrows. Amplicon stands for Sanger sequencing results from TA cloning, and more than one sequencing reactions were performed for amplicons of lncRNA00339 and FRILAIR. (TIF) [file pgen.1009461.s005.tif]

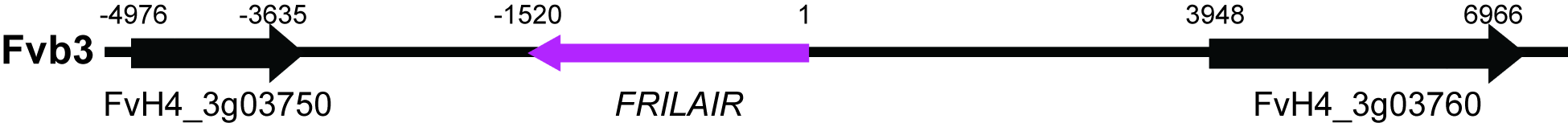

Supplement: S6 Fig — Pink arrow represents FRILAIR. Number above each lane stand for the distance to FRILAIR transcription start site. (TIF) [file pgen.1009461.s006.tif]

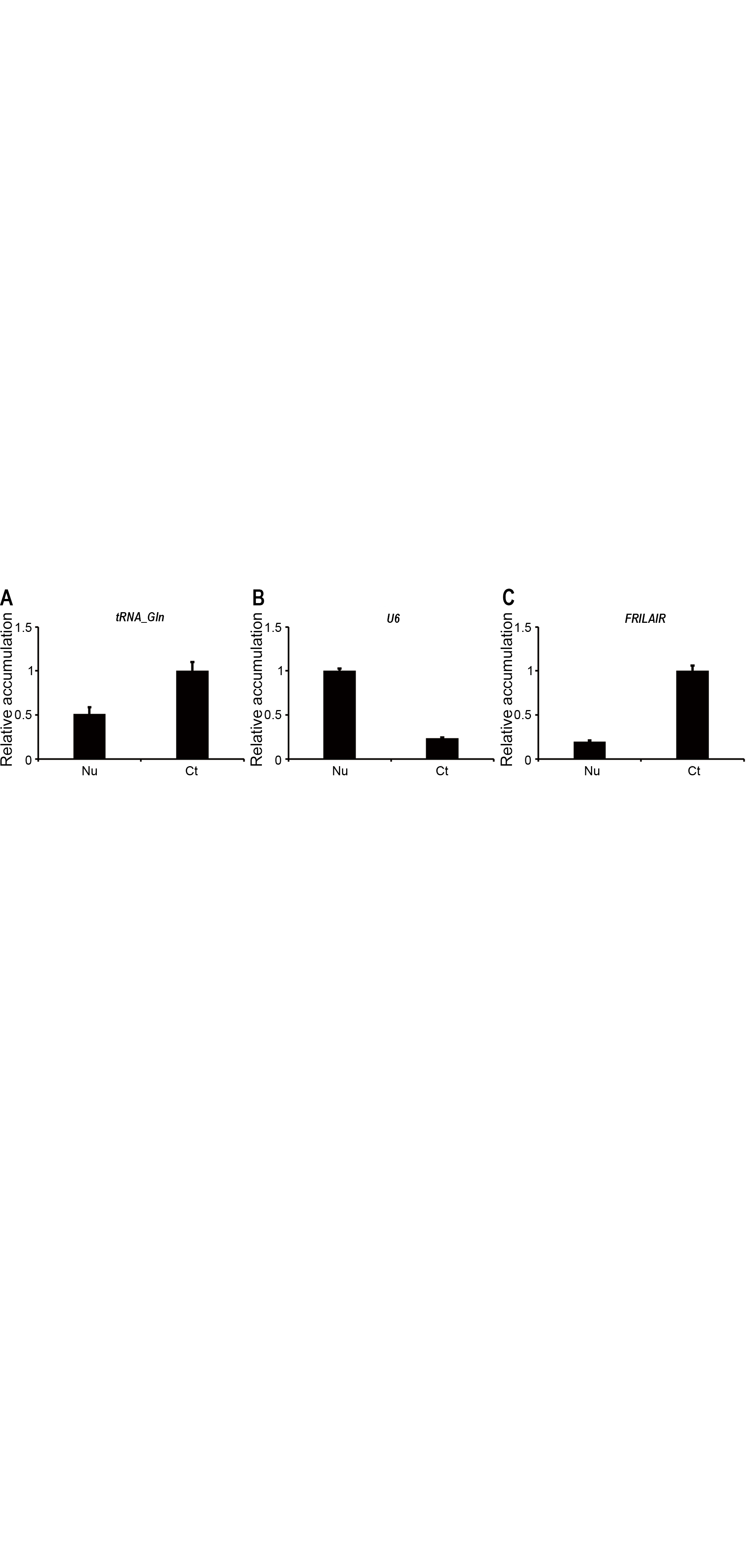

Supplement: S7 Fig — (A) Glutamine tRNA as the cytoplasmic RNA control (relative value set to 1 in cytoplasmic fraction), (B) U6 RNA as nuclear RNA control (relative value set to 1 in nuclear fraction), (C) total FRILAIR mRNA. Nu, nuclear fraction; Ct, cytoplasmic fraction. (TIF) [file pgen.1009461.s007.tif]

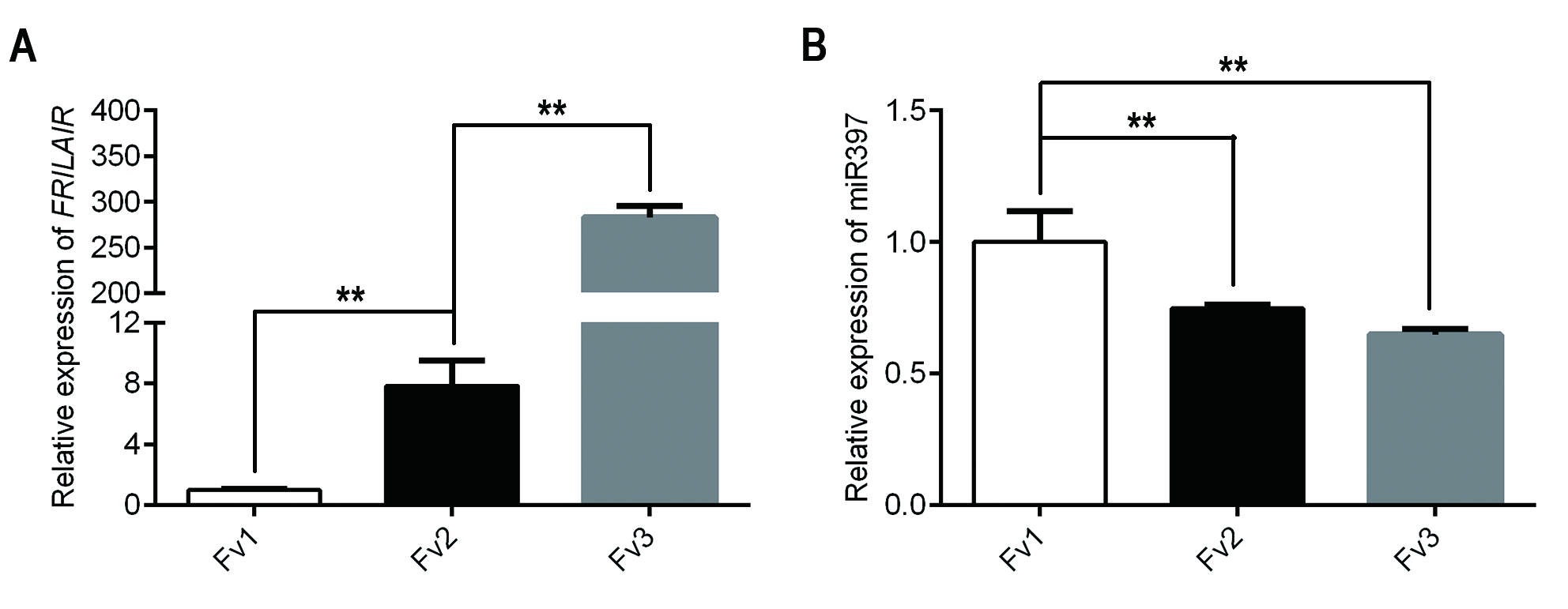

Supplement: S8 Fig — (A) Expression pattern of FRILAIR in strawberry fruit at three developmental stages in F. vesca. GAPDH was used as the internal control. (B) Expression pattern of mature miR397 in strawberry fruit at three developmental stages in F. vesca. U6 was used as the internal control. Statistically significant differences from control were determined by Student’s t-test: *P <0.05; **P <0.01. Values are means ±SD of three biological replicates. (TIF) [file pgen.1009461.s008.tif]

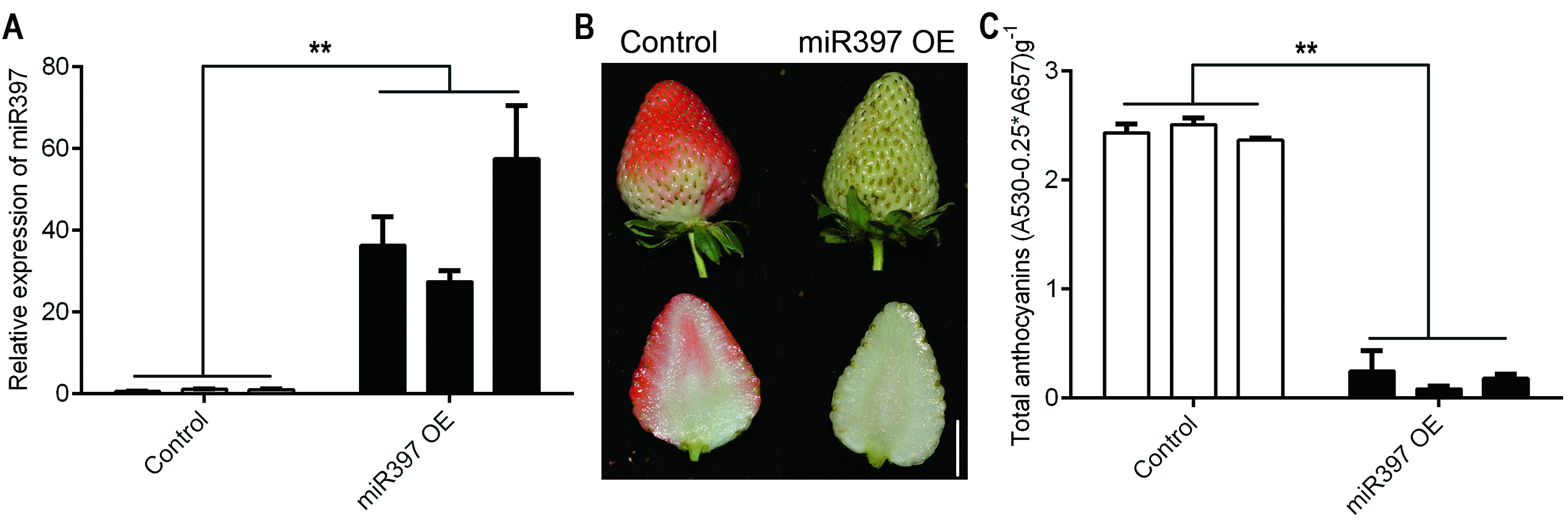

Supplement: S9 Fig — (A) qRT-PCR analysis of miR397 expression in miR397OE fruits. Agrobacterium tumefaciens-mediated transient transformations were performed on immature Falandi fruits at the big green stage, and strawberry fruits transformed with empty vector were used as control. U6 was used as the internal control. (B) Phenotypic analyses of fruits from miR397 OE. (C) Total anthocyanin content in fruits from Control and miR397 OE. The analysis was conducted five days after infection. Statistically significant differences from control were determined by Student’s t-test: **P <0.01. Values are means ±SEM of three biological replicates. Scale bar: 1 cm. (TIF) [file pgen.1009461.s009.tif]

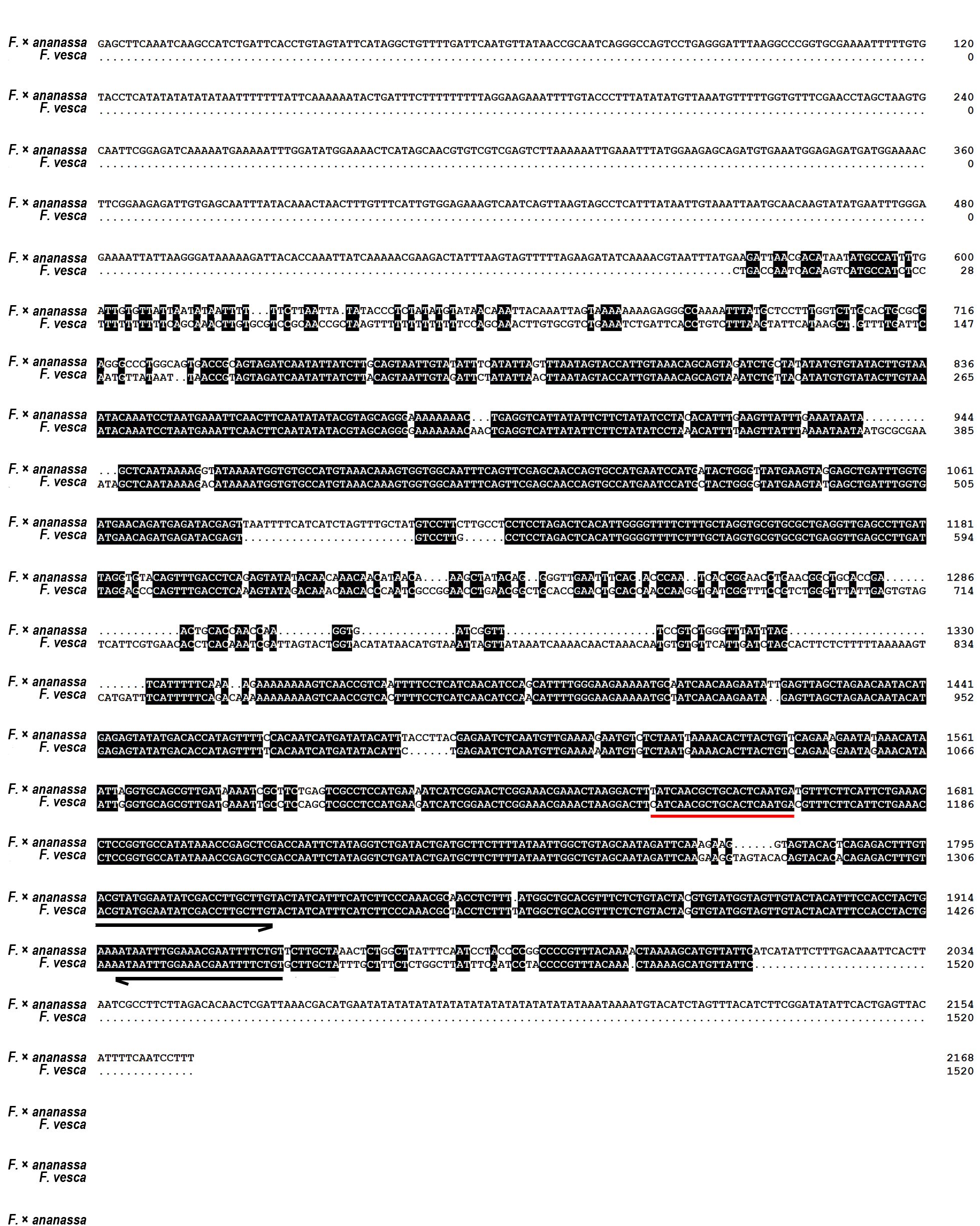

Supplement: S10 Fig — The red line indicates the miR397 target site of FRILAIR, and primers used in qRT-PCR are shown as black arrows. (TIF) [file pgen.1009461.s010.tif]

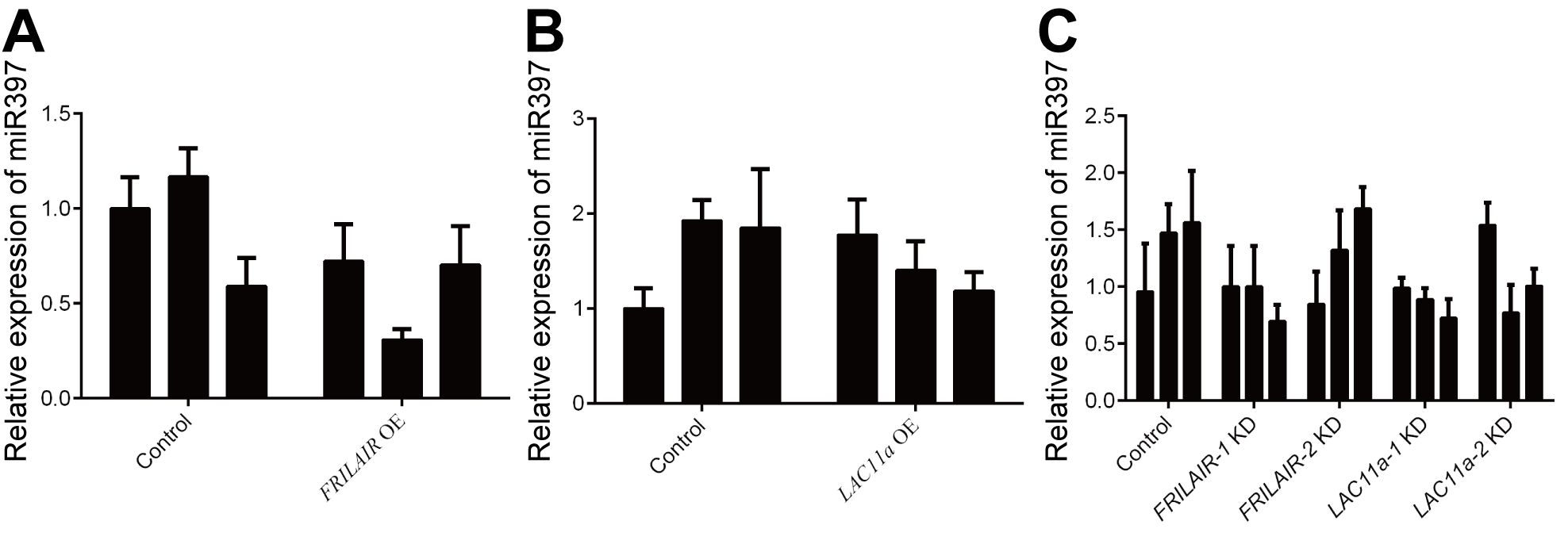

Supplement: S11 Fig — (A) Expression levels of mature miR397 in FRILAIR OE fruits. (B) Expression levels of mature miR397 in LAC11a OE fruits. (C) Expression levels of mature miR397 in FRILAIR KD and LAC11a KD fruits. U6 was used as the internal control. Values are means ±SEM of three biological replicates. (TIF) [file pgen.1009461.s011.tif]

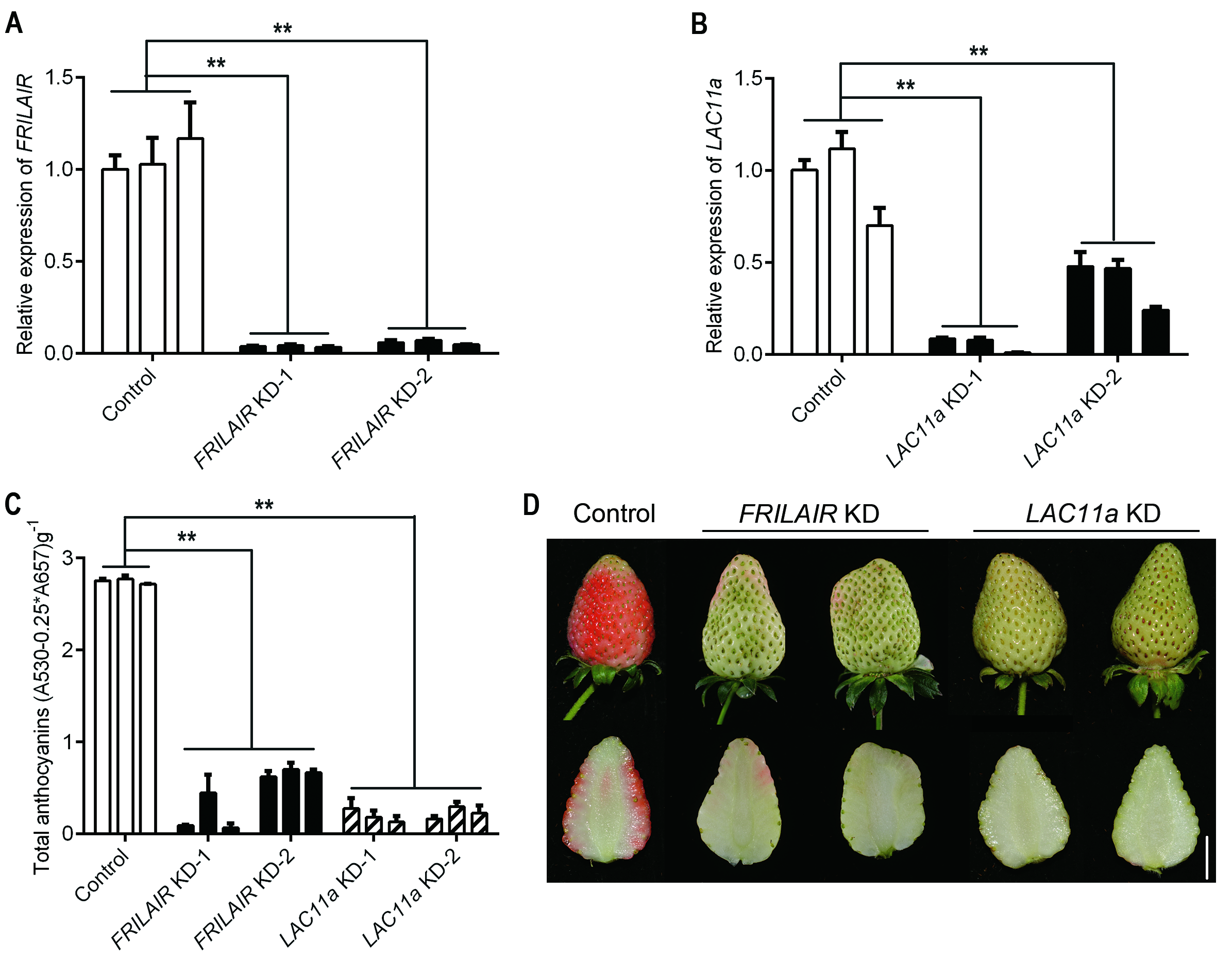

Supplement: S12 Fig — (A) qRT-PCR analysis of FRILAIR expression in FRILAIR KD fruits. Two independent sgRNAs targeted different regions of FRILAIR were applied to achieve two independent FRILAIR KD fruits including FRILAIR KD-1 and FRILAIR KD-2. (B) qRT-PCR analysis of LAC11a expression in LAC11a KD fruits. Two independent sgRNAs targeted different regions of LAC11a were applied to achieve two independent LAC11a KD fruits including LAC11a KD-1 and LAC11a KD-2. (C) Phenotypic analyses of fruits from FRILAIR KD and LAC11a KD. (D) Total anthocyanin content in fruits from Control, FRILAIR KD and LAC11a KD. Strawberry fruits transformed with pFveCas13b vector without sgRNA were used as control. GAPDH was used as the internal control. Agrobacterium tumefaciens-mediated transient transformations were performed on immature Falandi fruits at the big green stage. All analyses were conducted five days after infection. Statistically significant differences from control were determined by Student’s t-test: **P <0.01. Values are means ±SEM of three biological replicates. Scale bar: 1 cm. (TIF) [file pgen.1009461.s012.tif]

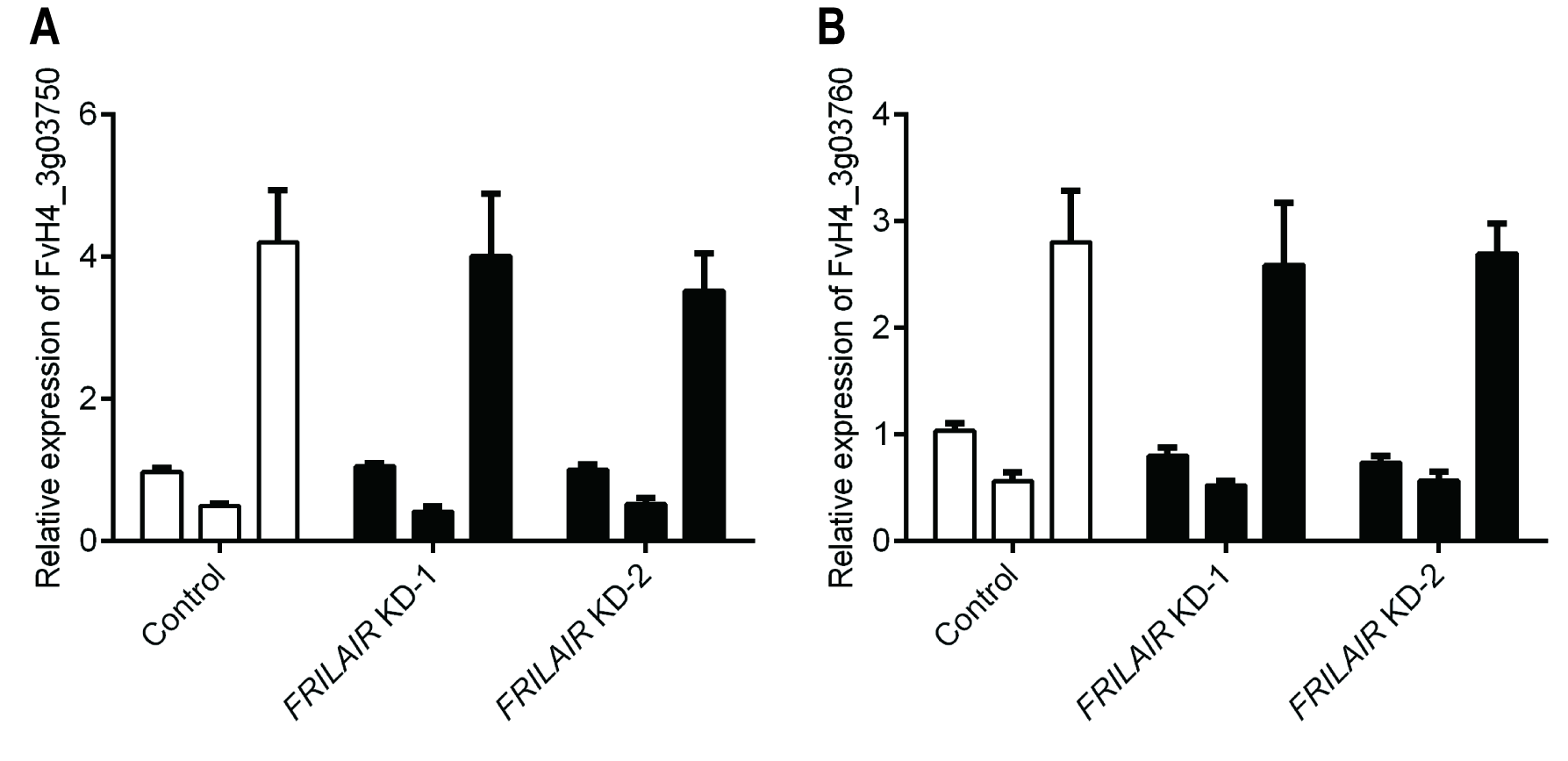

Supplement: S13 Fig — (A) Expression level of FvH4_3g03750 in FRILAIR KD fruits. (B) Expression level of FvH4_3g03760 in FRILAIR KD fruits. GAPDH was used as the internal control. Error bars represent SEM from three replicates. (TIF) [file pgen.1009461.s013.tif]

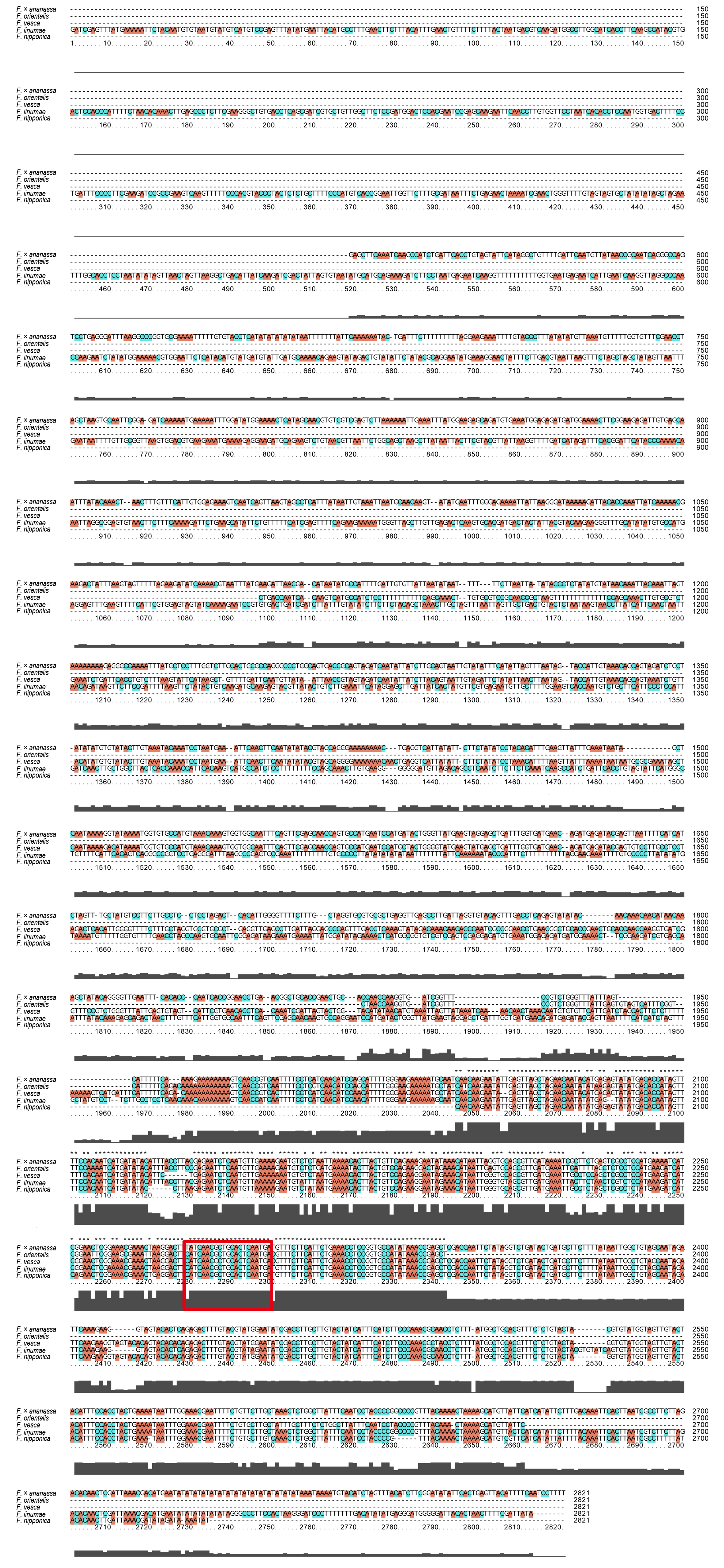

Supplement: S14 Fig — Sequence pairwise alignment was performed on FRILAIR from F. vesca, F. × ananassa, F. iinumae, F. nipponica and F. orientalis. The red box indicates the highly conserved miR397 target site. (TIF) [file pgen.1009461.s014.tif]

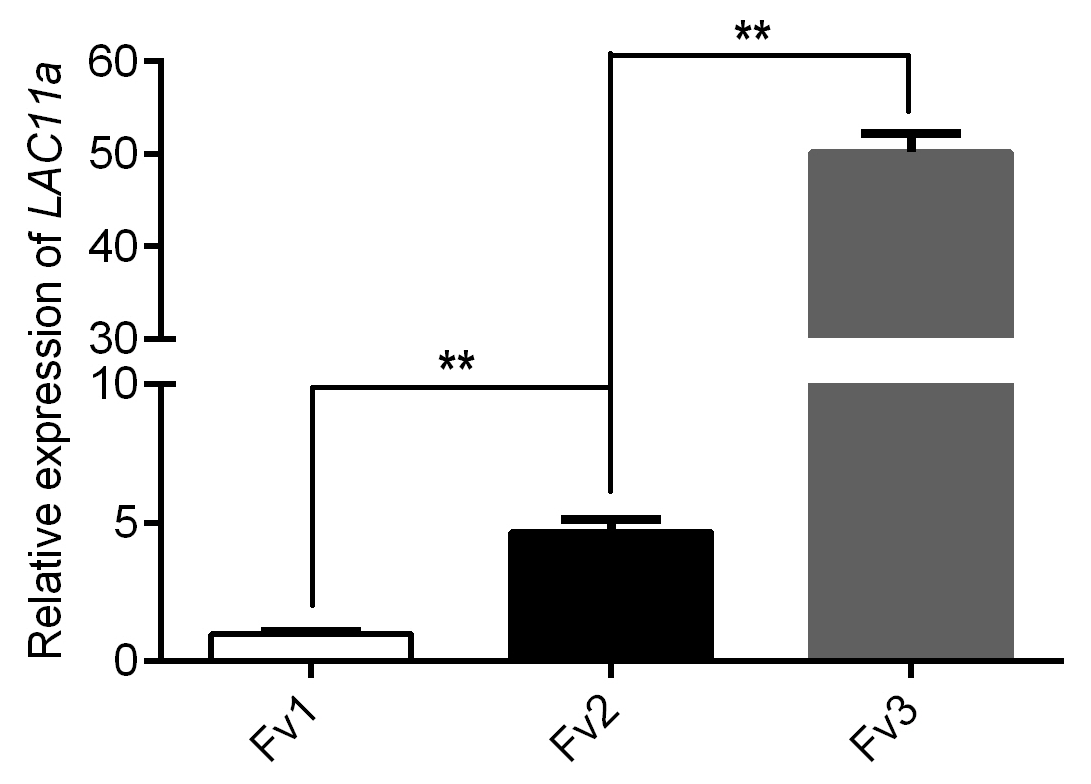

Supplement: S15 Fig — Statistically significant differences from control were determined by Student’s t-test: *P <0.05; **P <0.01. Values are means ±SD of three biological replicates. (TIF) [file pgen.1009461.s015.tif]

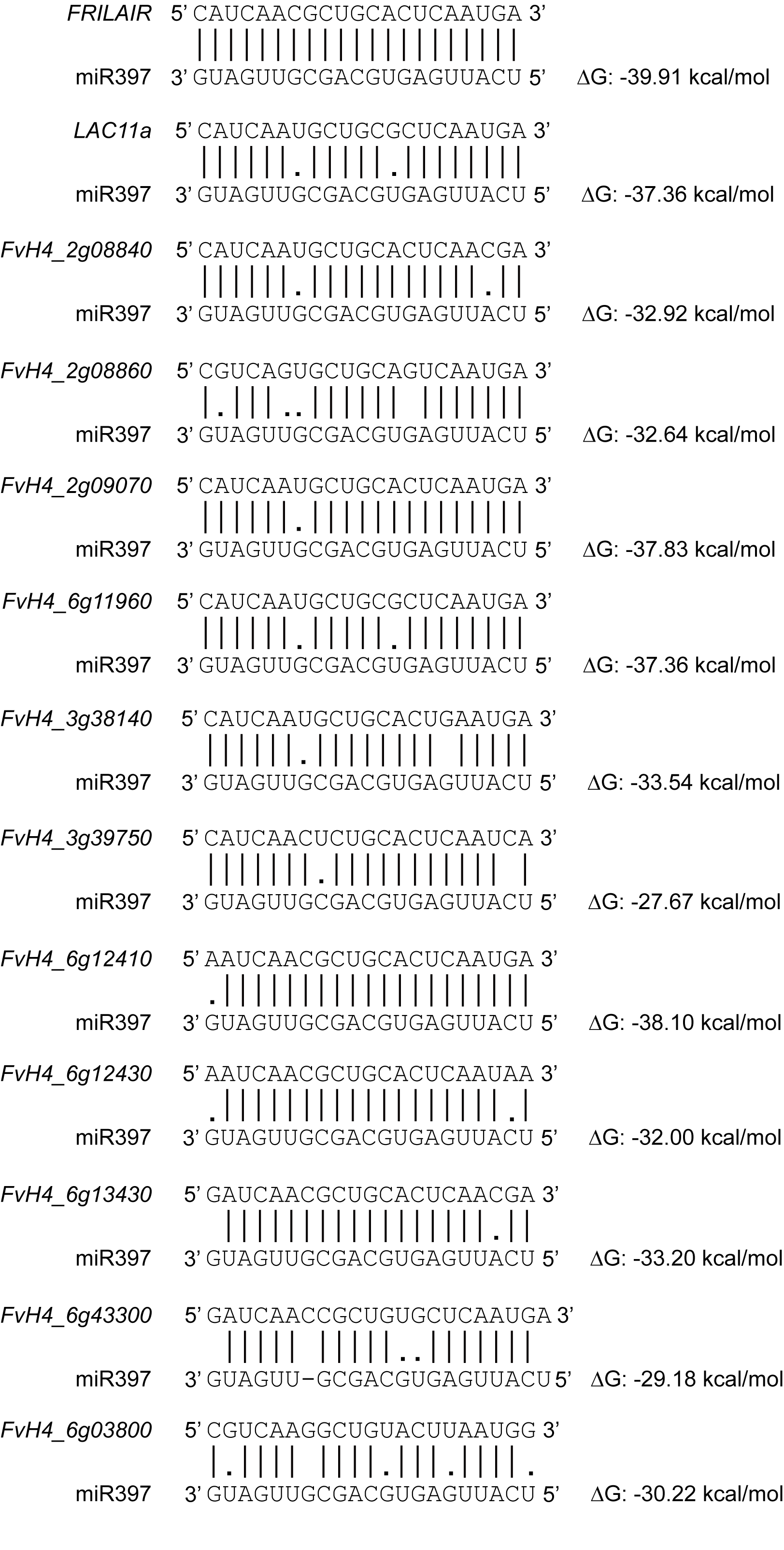

Supplement: S16 Fig — ΔG values were obtained from miRanda (Enright et al., 2003). (TIF) [file pgen.1009461.s016.tif]

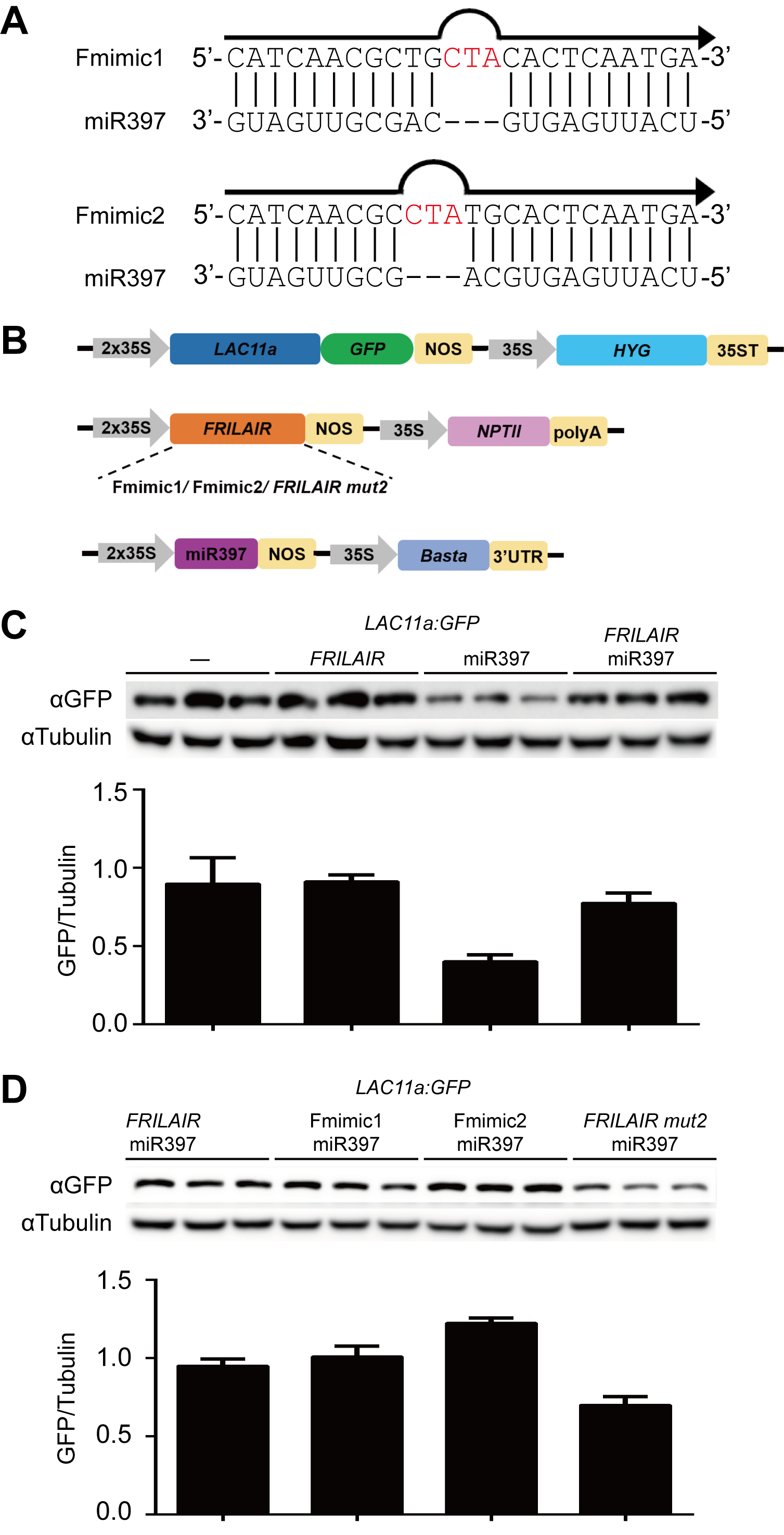

Supplement: S17 Fig — (A) Schematic diagrams of Fmimic1 and Fmimic2. (B) Diagrams of constructs used in the transient expression assay. (C and D) Transient expression assays in N. benthamiana, monitoring LAC11a:GFP by western blot. Relative LAC11a:GFP accumulation in the different agroinfiltration assays is indicated in bar graphs below each panel. Tubulin is shown as a loading control. Error bars represent SEM from three replicates. (TIF) [file pgen.1009461.s017.tif]

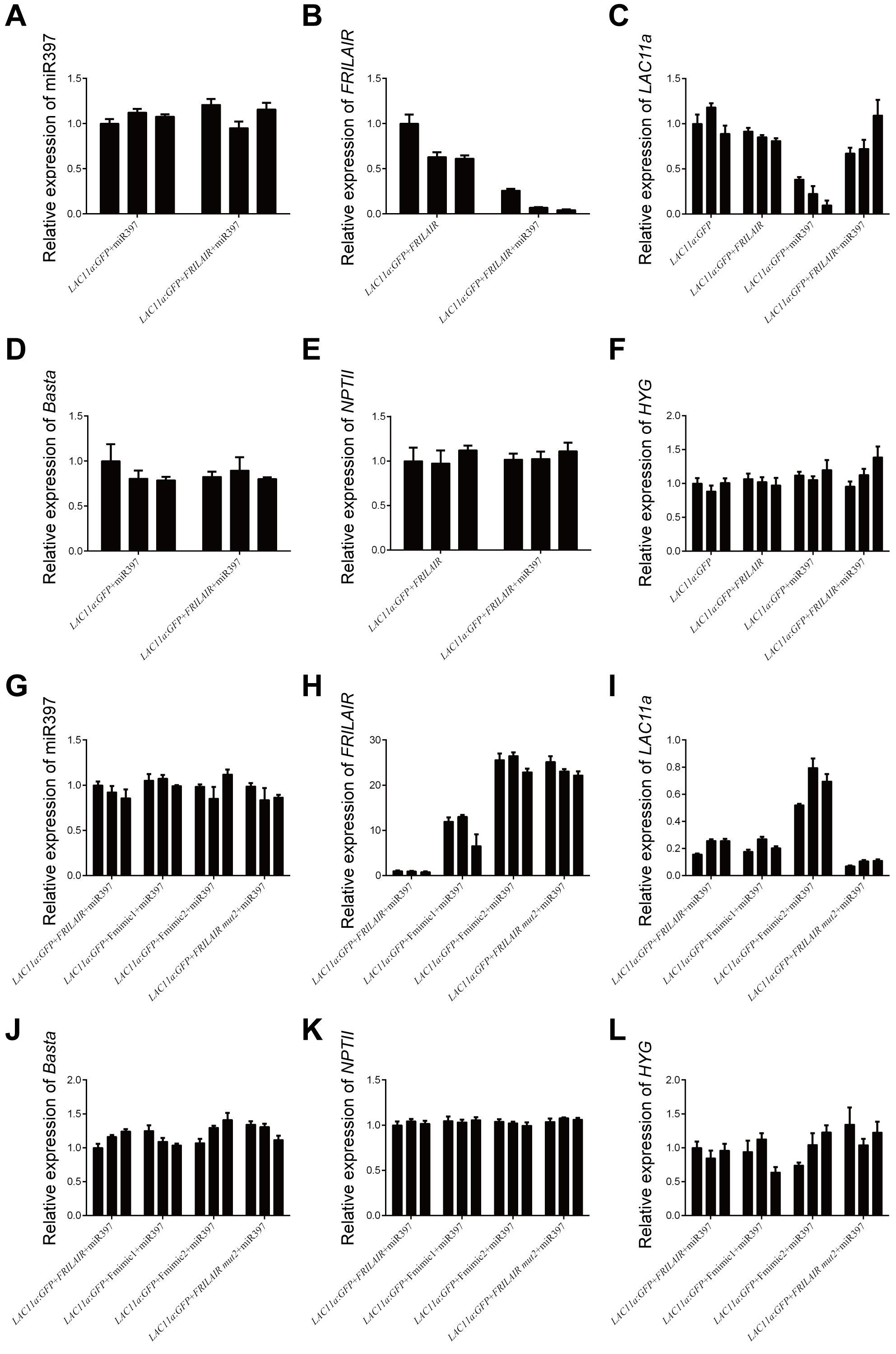

Supplement: S18 Fig — (A) Expression levels of miR397 in leaves of tobacco transiently expressed vectors including LAC11a:GFP + miR397 and LAC11a:GFP + FRILAIR + miR397 vectors. (B) Expression levels of FRILAIR in leaves of tobacco transiently expressed vectors including LAC11a:GFP + FRILAIR and LAC11a:GFP + FRILAIR + miR397 vectors. (C) Expression levels of LAC11a in leaves of tobacco transiently expressed vectors including LAC11a:GFP, LAC11a:GFP + FRILAIR, LAC11a:GFP + miR397 and LAC11a:GFP + FRILAIR + miR397 vectors. (D) Expression levels of Basta in leaves of tobacco transiently expressed vectors including LAC11a:GFP + miR397 and LAC11a:GFP + FRILAIR + miR397 vectors. (E) Expression levels of NPTII in leaves of tobacco transiently expressed vectors including LAC11a:GFP + FRILAIR and LAC11a:GFP + FRILAIR + miR397 vectors. (F) Expression levels of HYG in leaves of tobacco transiently expressed vectors including LAC11a:GFP, LAC11a:GFP + FRILAIR, LAC11a:GFP + miR397 and LAC11a:GFP + FRILAIR + miR397 vectors. (G-L) Expression levels of miR397, FRILAIR, LAC11a, Basta, NPTII and HYG in leaves of tobacco transiently expressed vectors including LAC11a:GFP + FRILAIR + miR397, LAC11a:GFP + Fmimic1 + miR397, LAC11a:GFP + Fmimic2 + miR397 and LAC11a:GFP + FRILAIR mut2 + miR397 vectors, respectively. Tubulin was used as the internal control. Error bars represent SEM from three replicates. (TIF) [file pgen.1009461.s018.tif]

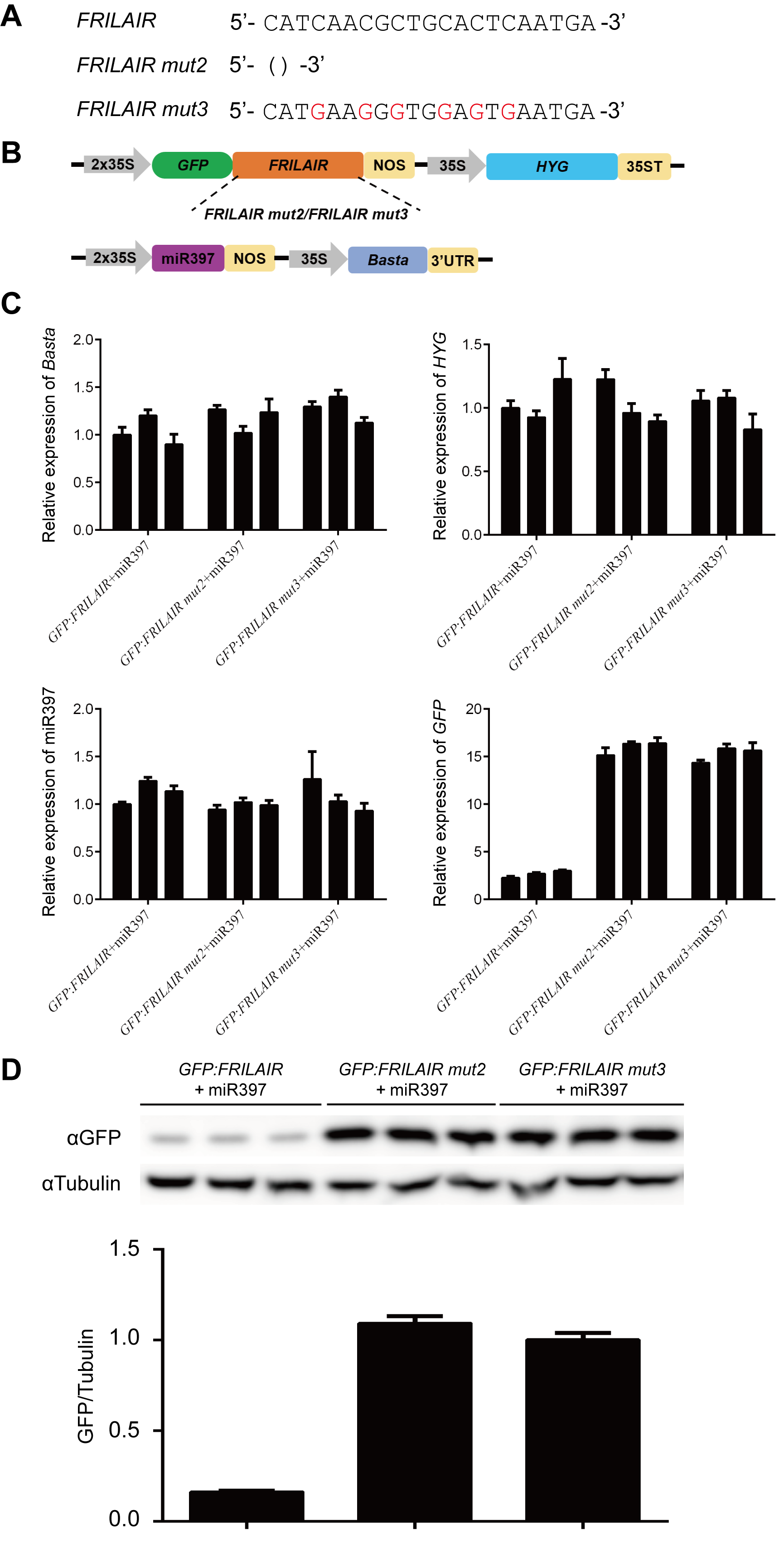

Supplement: S19 Fig — (A) Schematic diagrams of FRILAIR mut2 and FRILAIR mut3. (B) Diagrams of constructs used in the transient expression assay. (C) Expression levels of Basta, HYG, miR397 and GFP in tobacco leaves transiently expressing GFP:FRILAIR + miR397, GFP:FRILAIR mut2 + miR397 and GFP:FRILAIR mut3 + miR397 constructs. Tubulin was used as the internal control. HYG stands for the hygromycin resistance gene. (D) Transient expression assays in N. benthamiana, monitoring GFP:FRILAIR by western blot. Relative GFP:FRILAIR accumulation in the different agroinfiltration assays is indicated in bar graphs below each panel. Tubulin is shown as a loading control. Error bars represent SEM from three replicates. (TIF) [file pgen.1009461.s019.tif]

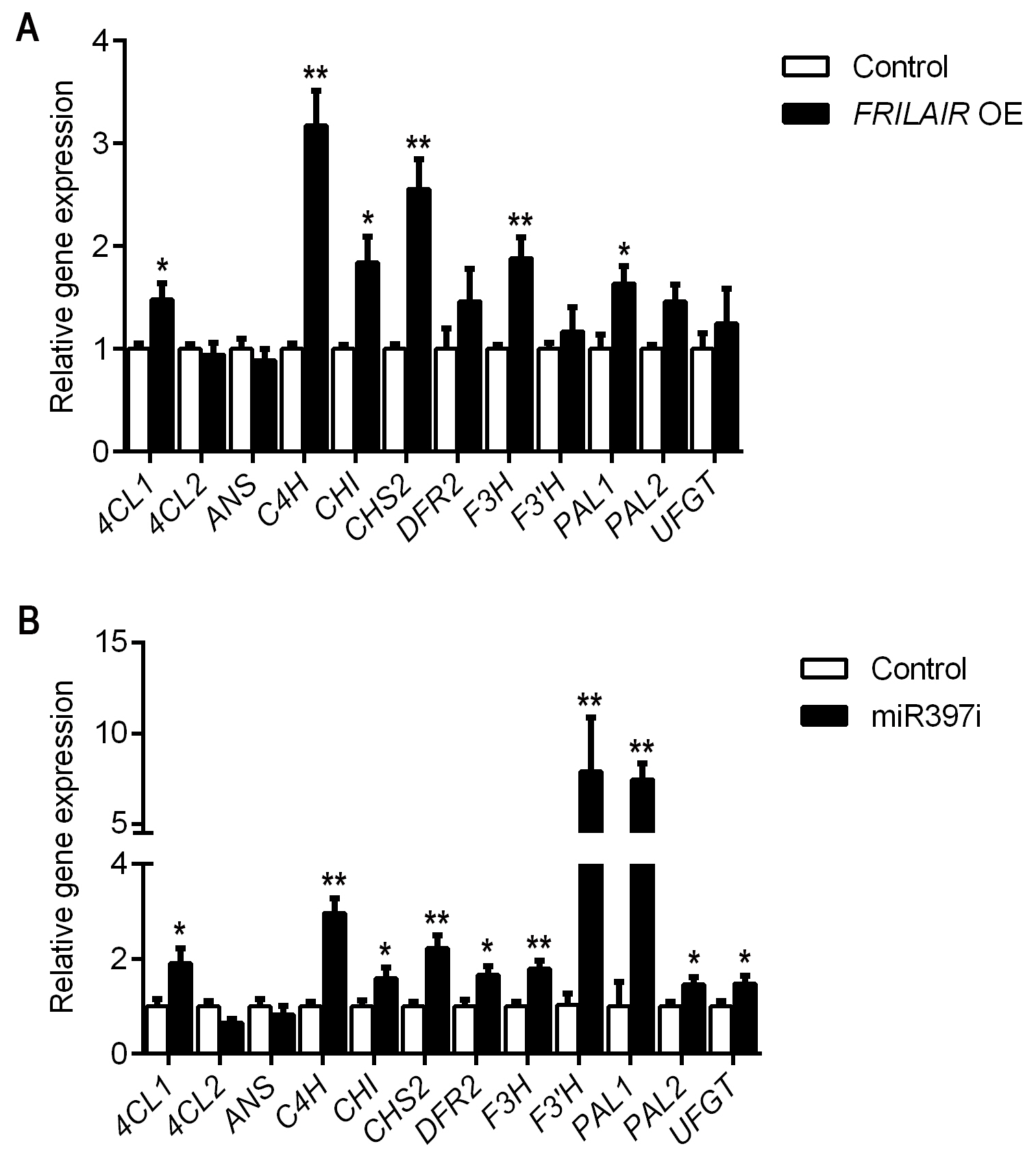

Supplement: S20 Fig — Relative expression levels of anthocyanin biosynthesis-related genes in fruits of FRILAIR OE (A) and miR397i (B). All analyses were conducted five days after infection. GAPDH was used as the internal control. Statistically significant differences from control were determined by Student’s t-test: *P <0.05; **P <0.01. Values are means ±SEM of three biological replicates. (TIF) [file pgen.1009461.s020.tif]

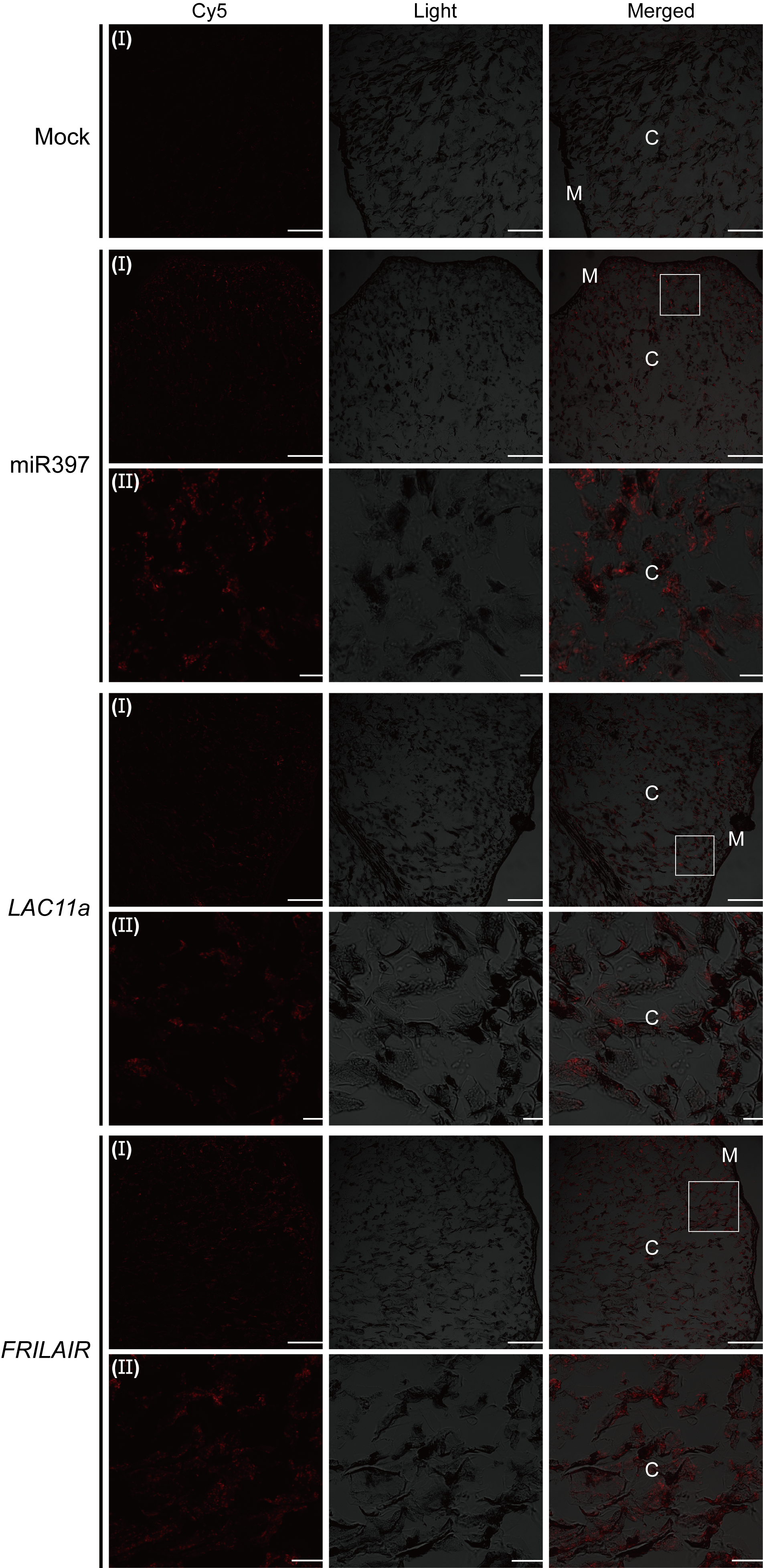

Supplement: S21 Fig — FISH was performed on horizontal sections of the cortex region. Images of (I) were observed with a Leica TCS SP8X confocal microscope under 10× magnification, and images of (II) were enlargements of the boxed area in (I) from the respective samples. Red fluorescence indicates the presence of corresponding RNAs detected by cy5-labeled riboprobes for miR397, LAC11a and FRILAIR, respectively. Mock hybridization used a cy5-labeled riboprobe that has no identity to the strawberry genome, and cy5 fluorescence signals were not observed. C, cortex region; M, margin region. Scale bars: 250 μm in (I), 50 μm in (II). (TIF) [file pgen.1009461.s021.tif]

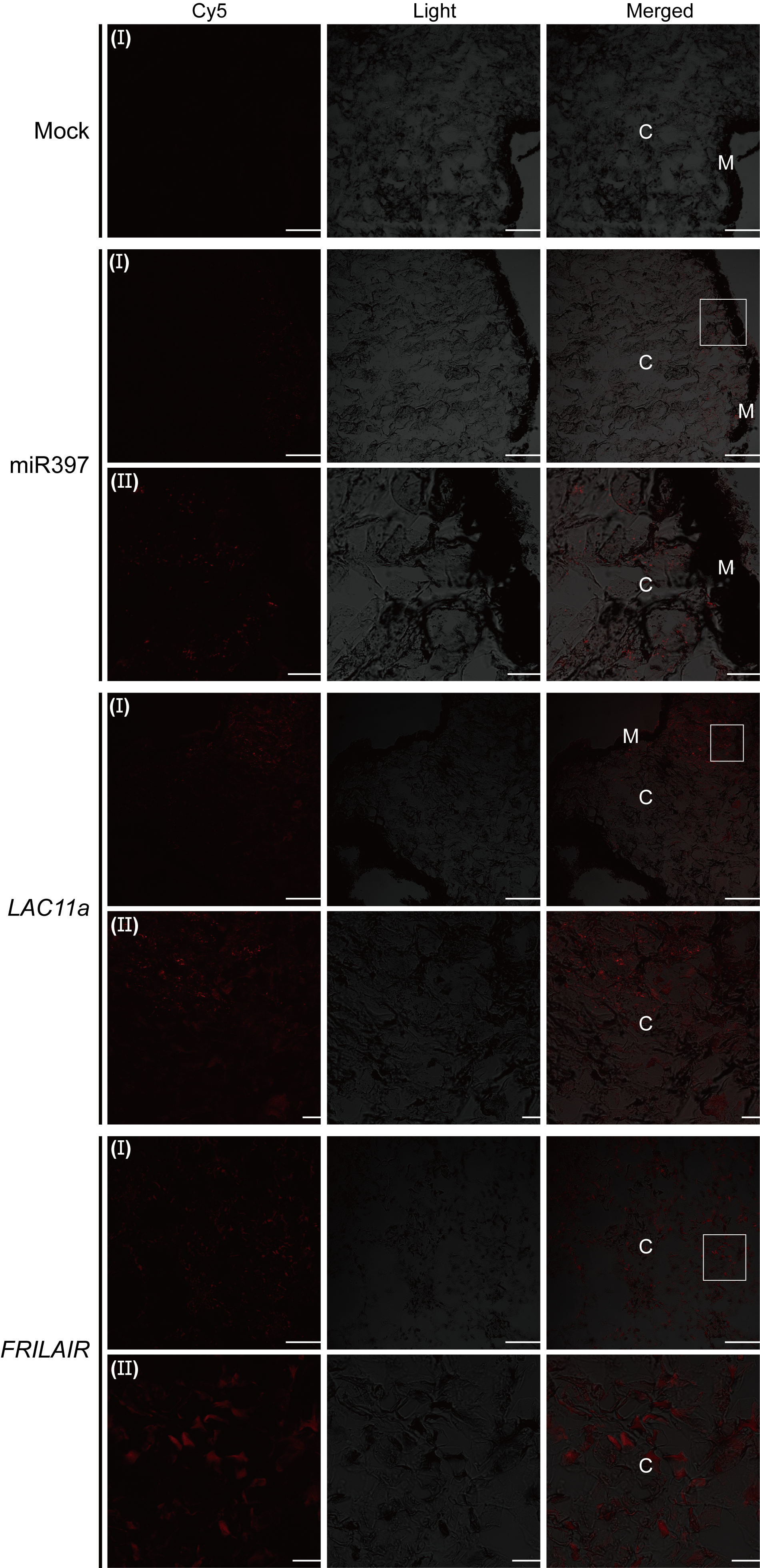

Supplement: S22 Fig — FISH was performed on horizontal sections of the cortex region. Images of (I) were observed with a Leica TCS SP8X confocal microscope under 10× magnification, and images of (II) were enlargements of the boxed area in (I) from the respective samples. C, cortex region; M, margin region. Scale bars: 250 μm in (I), 50 μm in (II). (TIF) [file pgen.1009461.s022.tif]
